# Spirit of the Foreign Periodicals, &c.

**Published:** 1836-04-01

**Authors:** 


### Spirit of the Foreign Periodicals, &c.

### Perforation of the Stomach, opening outwardly; the Patient lived for three Years.

Mad. G. aged 80 had, for the last 30 years of her life, been subject to repeated attacks of sharp, severe pain in the abdomen, which had caused her body to be bent forwards almost double. The functions of the stomach had been always more or less deranged, and frequently she had fits of vomiting of large quantities of glairy and bilious matter. The appetite however had been usually very good, and, at her advanced years, her strength was so preserved, that she was still able to walk about, and to attend to the economy of her small house.

In consequence of the habitual stoop or curvature of the body forwards, there was formed a permanent fold of the abdominal integuments, extending for about eight inches across the epigastric and hypochondriac regions. In June, 1832, the patient, being then 77 years of age, without having experienced any unusual pain or annoyance, observed, one morning, that the front of her chemise was quite wet; on removing it, a jet of clear water streamed out through a small aperture in the transverse fold just mentioned. Nearly two litres flowed out. An oozing continued for three days, and then it ceased spontaneously, and was not renewed. The abdominal pains, which had so long afflicted the patient, were much mitigated during several subsequent months; but they never entirely left her. For the following two years and a half, there were alternately periods of exacerbation and abatement; the intervals of ease gradually became longer, and, at length, her health seemed to be quite restored. In January, 1835, the abdominal pains returned, and, after continuing with greater or less severity for several days, the old perforation opened afresh, and a large quantity of clear fluid escaped. On the patient swallowing some port-wine and water, it flowed out almost as quickly as it reached the stomach, and the same was observed after "potage au vermicel" had been taken.

At this period Dr. Bineaux, the reporter of the case, visited the patient for the first time. The abdomen was free from pain and tenderness, except along the line of the transverse fold. There was no nausea, nor any other severe stomach distress. The perforation was of the size of an ordinary pea, rounded, its edges red, and it was situated in the epigastric region, rather on the left side, about two inches beneath the cartilages of the ribs. The surrounding parts were not hardened, swollen, nor otherwise diseased. This opening gave passage to any fluid the patient drank; and a quantity of gas also escaped. Occasionally, fæcal matters oozed out. The alvine and urinary discharges became greatly diminished in quantity, and the patient died on the 18th of the month. During Dr. B.'s attendance, the patient had not vomited once.

*Dissection.* The stomach contained a quantity of mucosity, and some semifluid aliment. The mucous membrane, throughout its greater extent, was healthy. Towards the pylorus, however, it was red, and its texture was considerably thickened. In the middle of the anterior surface of the organ, there was discovered a circular aperture, an inch and a half in diameter, having soft and smooth edges. The mucous surface here was but little discoloured. The aperture led into a cavity, from seven to eight lines in width; the sides of which were formed by cellular tissue, and the base by the portion of the abdominal integuments, through which the external opening had been formed. This opening did not exceed in width four or five lines.

The preceding case affords a beautiful illustration of the preservative effects of adhesive inflammation, after injuries or lesions of the internal viscera.

MM. Gerard and Lainé have published, under the auspices of MM. Chaussier and Percy, several examples of spontaneous perforation of the stomach. In most of them, the perforation was the result of a scirrhus or other morbid degeneration of the coats of the stomach.—*Archives Générales.*

The extraordinary and highly instructive case of perforation of the stomach, from a gunshot wound, recorded by Dr. Beaumont, and described at great length in our Number for January last, may be re-perused with advantage after reading the preceding details.—REV.

---

### ON THE EPIDEMICS OF INFLUENZA (GRIPPE.)

It is one of the most interesting themes of medical research, to examine the histories of various epidemics, with the view of ascertaining some of the more general facts or laws, as they are usually named, which relate to their appearance, dissemination, and departure. Such inquiries are of high practical import, not only by suggesting the most powerful means of counteracting the more immediate evils which these diseases inflict, but also by ascertaining the utility or inefficacy of quarantine restrictions in reference to them. That certain diseases are strictly contagious, or communicable by contact only, as several cutaneous diseases, syphilis, and, perhaps, we may add, the plague; that certain others are strictly infectious, or communicable only through the medium of the atmosphere, to those who are not far removed from the patients, as, for example, scarlatina, rubeola, cynanche parotidea, and, perhaps, also typhus fever; and that a third set, of which small-pox is an instance, may be propagated in both of these ways, and is, therefore, alike contagious and infectious;—these are facts which every medical man must admit. But the same degree of certainty does not belong to the characters of some other diseases, which, by one class of physicians, have been considered to be communicable by infection from one person to another, and, by another class, have been regarded as not at all infectious, but only as epidemically spreading, and seizing a number of persons at the same time, from the operation of the same widely-diffused agency. The history of the latest pestilence, which has brooded, like a bird of evil omen, over our own shores, as well as over those of almost every European country, we mean the Asiatic cholera, has made medical men attend more studiously to the important subject of epidemic influences. It will be remembered how patriotically active the contagionists of the orthodox Board of Health were at first—how copious and precise, and peremptory were the regulations (such, for example, as stopping all communication between the sea-port, where the scourge first shewed itself, and London by *sea*, and permitting the communication by *land* to continue!), which, it was believed, were to scare the monster from our isle, or to seize and hold him captive, if he dared to advance. WE laboured to disabuse these men of authority over us, and the public at the same time, of the fallacies they were committing, and of the vain hopes which they were cherishing; and, most assuredly, experience has amply confirmed all our predictions. Had the cholera been a less frightful malady, we are shrewdly of opinion that we should never have heard much of its contagiousness. But medical men are far too apt to be so mystified and terrified, by any thing new or uncommon, that they are frequently led into the most foolish admissions and commissions, until again set right by experience.

The history of the yellow fever of hot climates affords another apt illustration of the preceding remarks. But we shall not expatiate on this subject, but proceed at once to draw the attention of our readers to a brief notice of the leading invasions of a third epidemic disease. of not unfrequent occurrence. Like the other two already mentioned, the influenza has been supposed by some to be communicable, by infection, from one person to another. We believe that the only reason for this supposition is the circumstance of the cotemporaneous seizure of many persons with the same symptoms. But this is a most fallacious test, and in truth constitutes the essential character of an epidemic, rather than of a contagious disease. The invasion of the former is always more sudden, and its diffusion is much more rapid and more widely extended than in the case of the latter. Another distinguishing feature is, that a person labouring under an infectious disease may carry the communicable germ to a distance, and thus excite the disease in a place far removed from its original focus; but this is certainly not true of any epidemic disorder; and, lastly, we may point to the interesting feature of the one class conferring an immunity, more or less complete, against subsequent attacks,* whereas no such character belongs to the other class of maladies.

The term "la grippe" was applied in France to the catarrhal epidemic, (known in England by the name of the influenza,) in 1743, for the first time.

Before that period it was described as a "morbus catarrhalis;" an appellation too vague and general, and belonging properly rather to a class or set of diseases than to any one particular form.

It is more than probable that maladies very different from the "grippe" were comprehended under the denomination of morbi catarrhales, as we read "de febre catarrhali malignâ epidemicâ, anginâ gangrenosâ stipatâ;" a description which applies rather to malignant scarlatina, than to the genuine influenza. Whether the "pestilential and epidemic cough, which attacked almost the whole of Europe in 1580," was the genuine grippe, or not, we cannot now determine.

The derivation of the word "grippe," from the verb "gripper," to seize, to clutch, is disputed by Frank, who in his Prax. Med. Univers. Præcepta states, that its etymon is the Polish word chrypka, which signifies "hoarseness." The German writers call it the Russian catarrh, or the "lightning catarrh," blitz-katarrh, (from the suddenness of its seizure, and rapidity of its march,) and the Italians "mal del castrone" and "influenza;" the latter appellation being given to the disease from the supposed influence of the stars on its origin and diffusion.

The epidemic of 1743 invaded almost every country in Europe; and in many districts not a single family escaped its seizure. It broke out in the month of April, and disappeared in the following June.

It would be quite useless to detail its symptoms, as every man has seen cases of influenza, and the characters of one epidemic are very nearly the same as of every other. We observe that severe and obstinate headache was one of its most conspicuous symptoms. The same remark is applicable to the epidemic of 1833. "The treatment was generally an affair rather of diet and regimen than of any difficult medication;" an observation which is true of more epidemics than the one of 1743. In those days, as well as in the present, there were many practitioners, who were too busy and officious, unwilling to leave any thing to Nature.

The next general epidemic was that of 1762. It has been described by various authors under the names "morbus catarrhalis," "febris catarrhalis epidemica," "catarrhal disorder," "allgemeine brust-krankheit," (general disease of the chest,) &c. Again the severe head-ache is enumerated as one of the most troublesome symptoms.

The disease seldom proved fatal, except to old asthmatic people, and to those who had a tendency to pulmonary consumption. Many of the patients, however, retained for a length

* Not to allude to the extreme rarity of second attacks of variola, scarlatina, and other eruptive fevers, it is worthy of notice, as a fact which is not so generally known, that persons who have once had the plague, are in Egypt and Turkey considered to be not liable to its return. All the nurses and attendants in the lazarettos are "speste," i. e. "unplagued," or secure from the plague.

of time a feverish cough, and recovered their strength, but very slowly.

The epidemic of 1775 was not less widely diffused than its predecessors. It broke out towards the end of April, in almost every country of Europe, except England, where it did not make its appearance until the following Autumn. It was noticed at the time that the atmospheric vicissitudes which had been so remarkable during the year 1775, were considerably later in being experienced in our island, than elsewhere.

Very few persons escaped altogether the influence of this epidemic, and it was particularly noticed that it affected and modified the characters of other prevailing disorders.* Most of the domestic animals, as horses, dogs, and fowls, suffered more or less from the epidemic, being affected with cough, and general malaise. In the descriptions, and they are very numerous, left us of this epidemic, the critical reader cannot fail to notice the tendency to a continuance of severe local pains, sometimes in the head, at other times in the chest, loins, &c. for a length of time after the subsidence of the feverish symptoms. A full dose of the paregoric elixir at bed-time, and the use of mild tonics during the day, were the best antidotes to these sequelæ of the disease.

The next epidemic which deserves our notice is that of 1782. Before its appearance, the atmospheric changes had been very great. From cotemporaneous reports we learn that, previously to its invasion of Europe, it had been felt at Astrakan, Tobolsk, East Indies, and perhaps also in China. In January of 1782 it broke out at Petersburg. The thermometer had, in the course of one day, risen from 35° cent. below zero to 5° above it; and on that day, says Mertens, forty-thousand persons were seized with the influenza. In March it reached Poland, the shores of the Baltic and Denmark, and was known in these countries by the name of the catarrhus Russicus, or morbus Russicus. In May it passed over into Germany and England; in July it invaded Scotland and France; in September it visited Italy, Spain, and Portugal, and, in November, Belgium, which had hitherto escaped, was stricken with the distemper. But Asia and Europe were not sufficient to appease this insatiable monster; " elle parcourut non seulement toute l'Europe, mais encore toute la terre." America was not exempt from its passing dominion, and even the crews of ships at sea sickened from its pestilential miasm.

The epidemic of 1830 was quite as widely diffused as its predecessor. Like all the preceding epidemics it came from the East, and appeared after great alternations of atmospheric temperature. The headache was a very prominent symptom: it was usually confined, at least in an especial degree of severity, to the forehead and root of the nose, and sometimes was accompanied with an extremely sensitive or neuralgic state

* The influence which epidemics have on the type or characters of other diseases has been frequently remarked. The history of cholera, and that of some intermittent and remittent fevers furnish numerous illustrations. Dr. Marcé, speaking of an epidemic aguish fever which pervaded the department of the Loire-Inferieure in 1827, has the following remark. " Such was the influence of the reigning epidemic, that diseases the most different in their origin and symptoms assumed an intermittent and remittent character. A soldier, having exposed himself after violent exercise to a current of air, was seized with a tertian fever. An old man, who had met with an injury of the head, was affected with a 'soporose complaint having evidently a remittent character.' A coachman, who was labouring under stricture of the urethra, had an attack of well-marked ague after every introduction of the catheter.—(This is of not unusual occurrence in all seasons, healthy or not.)—*Rev.*

In the treatment of traumatic fevers, it was frequently necessary to have recourse to the use of sulphate of quinine, in consequence of their intermittent type."

of the integuments of these parts. The chief point of interest in the history of this epidemic is, the circumstance of its having preceded by a short interval, the appearance of another more formidable epidemic, we mean, the pestilential cholera. Between two and three years only elapsed, until the influenza again diffused itself over Europe. The numerous accounts in the various domestic as well as foreign journals furnish a most ample history of the epidemic of 1833, which seemed to follow the march of the cholera, advancing as its rival receded. We observe that the Italian doctors have indigenised the word "grippe" in their own soft musical language. Dr. Tinelli has communicated to the t. lxviii. of the Annali Universali a "Rapporto del Grippe che domino nella citta di Mantova."

Dr. Richelot has published the reports of several cases which he attended at the La Pitié; but the only allusion that we can make to them is to remark, that, in some instances, a painful swelling and enlargement of one or of both of the lower extremities supervened, as the feverish symptoms subsided.

If our memory serves us right, Dr. Tweedie has made mention, in his treatise on fever, of the occasional occurrence of such an accident in the decline of the disease; and we find that, so far as 1759, Dr. Hillary, in his Observations on the Dis. of Barbadoes, has written as follows:—"Towards the end of February, 1755, a number of persons were attacked with an epidemic catarrhal fever. In the greater number of the cases, one of the lower extremities became inflamed and much swollen, presenting the appearance of elephantiasis, but without any of the tumefaction in the groin, and the red streaks which usually extend from the groin to the leg in that disease."

In concluding his paper, Dr. Richelot has pointed out the leading features which are common to all the recorded epidemics of the grippe. They have all come from the east, travelling from the north-east towards the south-west: their attacks have been sudden, and in several parts of a city or district at the same time—their diffusion has been very widely spread—their duration short, and none of them has proved very destructive to life.

The influenza is truly a disease "sui generis;" it is essentially a constitutional or general disease, and not a mere "bronchite aigue," as some of the localising anatomical physicians of the present day would wish us to believe. The mucous membrane of the bronchi and trachea is, no doubt, irritated or slightly inflamed; but this affection is one only out of the many symptoms. It is unnecessary to allude to the treatment of a disease, which, in the majority of cases, may be left to the care of the kind nurse. When it attacks persons already suffering from illness, especially if the illness be seated in the lungs, the attendance of the skilful physician may save many a valuable life. Asthmatic and phthisical patients are liable to have all their distress greatly, and often alarmingly aggravated by an attack of influenza.—*Archives Gen. et Annali Universali.*

---

### On the Employment of Sheet-Lead, as an Application to Wounds and Ulcers.

M. Reveillé Parisé informs us that, when he was with the French army, at the famous siege of Saragossa, in 1808—9, the supply of medical stores for the wounded had been altogether expended, and that he was obliged to have recourse to a variety of substitutes for lint, plaster, and other dressings. Under these circumstances, he happily thought of using thin sheets of lead, which had the double advantage of being easily procured (by beating out common musket-bullets), and of being easily kept clean. Fortunately, too, this novel dressing was found to promote the cicatrization of many wounds. The plates must be as thin as a sheet of writing-paper, and uniformly smooth, especially where they are applied to raw surfaces. It may be supposed that they are apt to become uneven and wrinkled, and that thus they must rather irritate the wound or ulcer; but this objection applies rather to their misuse than to their proper employment. If they are secured in their place by bandages, well put on, it will be found that the lead-dressings are as smooth and comfortable to the patient's feelings as lint or any plaster. But it is not our present intention so much to recommend them to the exclusion of the ordinary applications, as to suggest their use as a most convenient and economical substitute on certain occasions. The expence of hospitals for the mere article of lint is very great; and, in time of war, it is not very easy to maintain a constant supply of it. The quality too of lint, in the present day, is exceedingly variable, a large proportion being uneven, knotty, and, from being bleached by the use of chlorine, apt to be irritating to the tender surfaces of wounds. When the discharge from a sore is scanty, the lint dressing often adheres to the edges of the wound, and there is difficulty in removing it; and, when it is more profuse, the lint is apt to form, at one part, quite a bag of matter, which is thus prevented from an easy escape. However indispensable, therefore, lint may be on the whole in the practice of surgery, it cannot be denied that it has its disadvantages, especially when the supply is not abundant, or the quality is not the very best.

The manufacture of this article has of late very sensibly diminished, in consequence of all the linen rags being eagerly bought up by the paper manufacturers, and also of cotton cloths having come so much more than formerly into general use; "le prejugé (adds our author) ridicule qui faisait regarder comme malsain de se servir de draps de coton, et d'envelopper les enfans dans les langes de cette substance disparait journellement." In consequence of the increased price of good lint, a variety of substitutes have been proposed. One of these is a fine sort of tow, prepared in a particular manner, and to which the name of "charpie vierge" has been given by the French surgeons; but the trials which have been made of this substance are quite unfavourable to its adoption. Should the lead plates be found by others to be as useful, as M. Parisé assures us they have been in his practice, he will have the merit of having introduced a very ingenious improvement into surgery. To prevent any misconception of his proposal, be it remembered that he recommends the use of the sheet-lead only to such solutions of surface as are "en voie de cicatrization," and not to recent or to spreading wounds. The ulcers to which the sheet-lead dressing is best suited, are those which are superficial and granulating, whose surfaces are covered with a layer of fibrino-purulent matter, and whose edges are red and even; in short, such as are healthy, and have a natural tendency to heal of themselves, if protected from all sources of external irritation. On the other hand, for ulcers which are deep, irregular, and painful, or of which the discharge is very profuse, we must have recourse to poultices, or other appropriate dressings, until the signs of incipient cicatrization make their appearance, and then the sheet-lead may be used with the best hopes of success. The large superficial wounds caused by some burns and vesicatories, especially in young children, are admirably suited to this mode of dressing. Most surgeons will acknowledge that these apparently simple sores have often baffled them most teasingly; when the ordinary applications seem to be useless or hurtful, recourse should be had immediately to the sheet-lead. It ought to be secured in its place by compresses and bandages, or by strips of adhesive plaster.

The softness and pliancy of the metal enable the surgeon to give the plate any shape he may wish, and to cut it as he would do a piece of paper. It is perfectly innocuous, the protoxide of lead being well known, under the name of litharge, as a common application to raw surfaces. We do not, by this remark, mean to imply that the lead plate acts in any other manner, than as a means of mechanical defence and support.

M. P. has tried sheets of tin, silver, and gold, and he has found them all succeed nearly equally well, provided they be made perfectly smooth, and applied so evenly and uniformly, that no irritation is induced; but, in point of ductility, the lead is greatly superior to tin, and its cheapness recommends it before the others.

That greasy applications are positively injurious to some ulcers, will not be disputed by any surgeon. How often do we observe that mere abrasions of the skin, situated over superficial bones, as the tibia, are obstinate and most troublesome of cure, and that all the ordinary dressings seem to do rather harm than good. If we apply dry lint, or use poultices or lotions, in the former case, the sore is apt to be fretted by the lint adhering to it, and in the latter, the disposition to heal often appears to be interrupted. Under such circumstances, the use of the lead plate will be found to be at once most simple and efficacious.

A gentleman, while descending a staircase, wounded his heel against the sharp edge of one of the steps: the wound was small, and quite superficial, and instead of healing in the course of a day or two, as was expected, it remained open for several weeks, in spite, or perhaps rather in consequence, of a variety of ointments and plasters, which were successively tried. The patient annoyed that so trifling a wound baffled so much "doctoring," dressed it of his own accord with dry lint; but this would not do; the lint stuck to the edges of the wound, and confined the discharge, and the sore became deeper and larger than ever. M. Parisé's advice was now taken. A piece of smooth and very thin sheet-lead was laid on the wound, and kept in its place by two or three slips of plaster. Under this simple dressing, repeated for several days, the wound very speedily healed.

Every surgeon must have experienced occasionally a most troublesome delay in curing ulcers, or indeed simple abrasions of the skin over the olecranon, spine of the tibia, malleoli, &c. As a general rule, we should dissuade the use of greasy or adhesive plasters, or even of dry, or wetted lint in such cases; and advise the patient merely to cover the injured part with a piece of sheet-lead, or of any other equally simple and innocuous application, such as goldbeater's skin, and leave the cure altogether to Nature.

One advantage of the sheet-lead over the usual applications, to which we have not alluded, is, that there is less necessity to renew the dressings so frequently, as when lint is used; for, the wound is kept much cooler, and the tendency of the discharge in hot weather to become acrid and offensive, is very sensibly diminished. There is one form of superficial ulceration, which is very often most difficult and obstinate of cure; we allude to herpetic and impetiginous sores. Now, although we do not mean to assert that the sheet-lead is adapted to every sore of this description, or that it has any healing efficacy *"per se,"* we do not hesitate to recommend its use, in preference to the ordinary applications. Ointments, and all greasy or irritating plasters, very generally disagree with these sores; and, on the whole, simple astringent washes appear to be most serviceable. The chief objection to the use of these is, that the linen, which has been wetted and applied, is apt to become occasionally dry, and to adhere to the raw surface, and thus to irritate it and prevent it from healing. The sheet-lead prevents the rapid evaporation, and thus keeps the sore in a moist state during the intervals of the wet application.

The sores which are apt to occur on œdematous limbs is another tribe which is often equally intractable, and which very often appears to be rather aggravated than relieved by the ordinary dressings. M. P. has had recourse to the use of mildly stimulating washes, and the sheet-lead with decided advantage, in a number of such cases.—Baron ——, advanced in years, had for some time been labouring under chronic hepatitis, which had at length induced ascites and general anasarca.

The distended skin of the legs became red, tender, and excoriated, in consequence of the friction of the bedclothes, and of the bandages with which the limbs were swathed. The excoriations were at some places so deep that they might be considered rather as ulcers than as mere abrasions of the skin.

The finest charpie, the mildest washes and ointments, were tried; but every means appeared only to aggravate the distress. M. Parisé being now summoned to his assistance, recommended that the limb should be kept in the horizontal position, the raw and ulcerated surfaces be covered with very thin sheet-lead, and this to be secured by a few turns of a linen roller. The comfort derived from this simple treatment was speedy and decided, and the sores began gradually to cicatrise and heal up. During the remainder of the Baron's life, there were frequent returns of his dropsical complaint, and at these times, the limbs always suffered in the above-mentioned manner. The same local treatment was adopted, and invariably with success.

As an example of the effects of the sheet-lead dressing in the ulcerated surfaces which are apt to follow the application of blisters in some constitutions, and especially in young children, we quote the following case.

M. P. was requested to visit a child, on whose arm a blister had been applied for an obstinate chronic bronchitis. The raw surface had never shewn any disposition to heal; it had become ulcerated, and had extended itself in all directions, so that nearly the whole arm, from the shoulder to the elbow, was excoriated and inflamed. The child's health had suffered much from the constant pain, and irritation, and want of sleep. The dressings, which had been used, had been either utterly useless; or positively injurious. M. P. recommended that the arm be covered with a piece of thin sheet-lead, and no other application be used. In the course of a very few days, the whole of the raw surface was completely healed.

The sheet-lead is well adapted to most varicose ulcers. As a matter of course, regular and even compression of the limb must be employed at the same time. Professor Marjolin has recommended this treatment for such sores, in the article, Ulcere, Dict. de Medecine.

In conclusion, we may confidently recommend the use of sheet-lead as a dressing to all ulcers, which are superficial, and from which the purulent discharge is not very profuse; more especially when they have a tendency to cicatrization, and when, therefore, it is the principal object of a surgeon to defend them from any irritation which may interrupt the healing process.—Even when the suppuration is abundant, some practitioners have had recourse to the sheet-lead, with the simple modification of pricking a number of holes through the thin plates, for the purpose of allowing part of the matter to ooze out. The œconomy, cleanliness, and convenience of the remedy, as well as the avoidance of all irritation, itching and other distress, which are so frequently induced by the common dressings in certain ulcers, are such obvious advantages of the sheet-lead used as we have explained in the preceding observations, that they cannot fail to recommend it strongly to the attention of the practical surgeon.—We have only further to state, that the sheets or plates ought to be made of different thickness, and that they must be always perfectly smooth, even, and of such consistence, that they may be readily cut into any size and shape with a pair of ordinary scissors.—*Bulletin Generale de Therapeutique.*

When it is the wish of a surgeon to apply to a wound or excoriation an adhesive plaster, which is devoid of all acrimony, he will find none more useful than one made by washing a piece of oiled silk with a strong solution of isinglass in alcohol. The solution is promoted by immersing the vessel containing it in hot water. The common adhesive plaster, and the black court-plaster, often teaze and irritate a raw surface. The isinglass plaster requires to be slightly moistened before it is applied. Mr. Liston has the merit, we believe, of having first recommended it.

---

ON THE TREATMENT OF ERECTILE TUMORS. By PROFESSOR LALLEMAND, of Montpelier.

CASE 1. *Erectile Tumor of the Gums and Lower Jaw.—Cure by Excision.*

A boy, nine years of age, had a fungous tumor, situated on the gum of the lower jaw, extending from the right incisor teeth round to the first left molar tooth: it was indolent, of a purplish colour, mamillated, and felt soft and somewhat fluctuating to the finger.—The slightest pressure caused it to become smaller and paler, but on withdrawing the pressure it immediately recovered its former size and colour. The lower lip opposite to the centre of the tumor was of a violet red hue, and was traversed with numerous veins, much enlarged, as if they were varicose. Various attempts to remove the disease by excision and cauterisation had been ineffectually made during the preceding two years: the tumor had always quickly sprouted up again. M. Lallemand intended at first to have removed the entire substance of the lower jaw, where the disease was seated; but finding that the base of the bone was quite healthy, he was anxious to save as much as he could, in order that the continuity of the jaw might not be broken.

The lip being dissected back from the jaw, he removed every portion of the tumor with the knife; then applying the crown of the trephine, he endeavoured to saw off the anterior alveolar plate of the upper two-thirds of the bone; but this he could not easily effect, in consequence of the instrument coming into contact with the new teeth, which had not yet been protruded. After removing as much as he could with the "le trepan exfoliatif," he finished this part of the operation with the gouge and mallet. The actual cautery was then freely applied to every suspected point. The wounds of the soft part were brought and kept together by means of twisted sutures, and by bandages. The result was very fortunate. Union had taken place at the end of a week. Ten days later, a sequestrum of bone exfoliated, and in the course of another week, the cure seemed to be complete. In course of time, the new teeth, which had been exposed by the trephine, continued to advance; the canine exhibited the prints of the instrument.

M. Lallemand strongly urges on the attention of surgeons the propriety of endeavouring to save as much of the lower jaw in such operations, as may be done with safety. It is not common that the entire thickness of its substance is diseased; the mischief is generally confined to the anterior alveolar lamella.

Case 2.—*Erectile Tumor on the Upper Lip and the Edges of the Nostril. Cure by numerous Incisions, and by the use of the twisted Suture.*

A child, five years of age, had been born with a red spot, of the size of a pea, on the centre of the upper lip. It had extended gradually, and had involved first the septum narium, and then the inner surface of the nostrils.

On his admission into the Hospital St. Eloi at Montpelier, the upper lip was red, and so much swollen, that it hung down over the lower one: the slightest excitement caused it to become distended and to throb strongly, and in other respects it exhibited all the characters of an erectile tumor.

To treat this case by compression, was impracticable; excision of the disease would have caused a most frightful deformity; and the chances of success, by tying the trunk of the labial, or of the external carotid arteries, were very unsatisfactory. M. Lallemand tried a new method. He excised from the middle of the fungus a triangular segment, (from eight to ten lines in breadth at its base, and pointing to the septum narium,) by means of sharp scissors. The blood streamed out with violence from a multitude of divided vessels, and the lip quickly recovered its normal size and colour. The edges of the wound were laid hold of, and compressed by assistants, and M. L. proceeded to unite them by means of a twisted suture; four needles having been inserted at some distance from the wound, and made to traverse the whole extent of the diseased part. The operation was finished, as in cases of hare-lip. The hæmorrhage was completely arrested.

Considerable febrile excitement and local distress came on, but in the course of two days, these symptoms subsided. On the fourth day, the two uppermost needles were withdrawn, but the coils of thread were not removed, and, on the following day, the other two needles were withdrawn. There was a trifling oozing of blood and matter from the small wounds, caused by the needles. At the end of the third week the threads separated, and exposed a firm and healthy cicatrix, extending from the centre of the lip to the septum narium.

Having obtained from this operation the result which he had hoped for, M. Lallemand repeated it on that part of the swelling situated between the cicatrix and the right commissure of the lips. In passing the needles through, it was found that there was considerably more resistance on the inner (*i. e.* nearest the cicatrix) than on the outer side. This circumstance indicated that the structure of the part had become agglutinated and hardened, in consequence of the inflammation induced by the previous operation.

A similar operation was subsequently performed on the left side, between the cicatrix and the left commissure of the lips. The results were quite as fortunate as on the former occasions. The lip, in course of time, recovered its normal size, and it no longer hung down over the lower one; all the parts involved in the operations were converted into "une espece de fibro-cartilage." The septum narium and the mucous lining of the nostrils remained, however, still tumefied, and M. Lallemand therefore, determined to pass several needles through the septum, for the purpose of exciting an agglutinative inflammation. In doing this, he found that the median cicatrix was much more firm and resisting than even the cartilage of the septum.

The ultimate success of this case was highly gratifying to the surgeon, who, by a simple and judicious practice, had saved his patient from the suffering, as well as the danger, of the more formidable operation of excising the tumor.

CASE 3.—*Successful Treatment by Incision and the Twisted Suture.*

A child, three months and a half old, was brought to M. Lallemand for advice respecting an erectile tumor, situated on the left cheek; it was about two inches long, and one and a half broad, and it projected nearly half an inch above the adjacent surface. It became larger, and of a more purple hue whenever the infant cried, and exhibited then quite the appearance of a large strawberry. This tumor had increased very rapidly from the birth, at which period it was only about a quarter of an inch in diameter, and not at all prominent. The impracticability of exercising a continued compression, and the risk attending an attempt to cut the diseased part out, induced M. Lallemand to repeat the operation which had succeeded so well in the preceding case. He made an incision through the entire thickness of the swelling, and in the line of its longest diameter; he then immediately united the edges of the wound with four needles, and secured these by numerous coils of thread, as in the operation for harelip. On the following day, the parts which were exposed were very painful, much distended, and of a deep purple colour. On the fifth day, the needles were withdrawn. At the end of a fortnight, a tolerably firm cicatrix was formed along the lines of the incision and of the punctures, and the rest of the swelling had much less of the purple hue which it had exhibited before. In four months, all that remained of this erectile tumor was a few red scattered points round the edges of the once diseased part. The skin of the cicatrix was still thin and shining, as after a burn.

CASE 4.—*Erectile Tumor on the Shoulder—Cure by the Twisted Suture, without Incision.*

The child was born with a red-coloured spot, of about an inch in diameter, on the skin covering the left scapula. It rapidly increased, so that, in the course of three months, it measured at least three inches one way, and two another, and it had become considerably prominent above the surface. Its surface was mamillated, and of the colour of a ripe strawberry. Graduated compression had been tried for several weeks, but with no benefit. Afraid of the excessive hæmorrhage which might follow the excision of this tumor, M. Lallemand decided to adopt a treatment somewhat similar to what had succeeded so well in other cases. Without making an incision through the diseased part, he merely passed twelve needles through its lower part, and "couvrit l'espace qui les separait par des circonvolutions nombreuses de fil ciré." Three days afterwards, he performed a similar operation on the upper part of the swelling. In this manner, he attacked successively the whole of its circumference, always leaving the needles in for about eight days or so, until an agglutinative inflammation had been fairly excited.

By the end of four weeks, all the circumference of the tumor, being either "affaissée," or in a state of suppuration, M. Lallemand intended to repeat the operations on its central part; but, most fortunately, a process of inflammation had already commenced there, and, in the course of a fortnight, the entire extent of the diseased substance was converted into a flat and uniform cicatrix. The treatment of this case lasted for nearly three months. During that period, 120 needles had been successively passed through different points of the tumor, and yet not one spoonful of blood had been lost, nor had the general health of the patient suffered for more than three or four days in all.

The case now detailed is interesting in an especial degree, from the circumstance of the cure having been effected by acupuncturation alone, without the previous step of incising, or partially excising the tumor, as had been practised in the two cases immediately preceding.

M. L. states that he now considers the incision to have been unnecessary in the third case; and that, in a similar example, he should recommend the use of the needles alone. The removal of the triangular portion of integuments practised in the second case, he also deems to have been uncalled for—a simple incision through the diseased structure would have answered as well.

The use of the ligatures may be necessary, and indeed ought to be adopted, whenever we intend to incise an erectile tumor; but, when we do not, there is, perhaps, not much occasion for them. M. L. alludes to the utility of frequent applications of the nitrate of silver to the minute wounds left after the withdrawal of the needles.—*Archiv. Générales.*

---

### Influence of Hypertrophy of the Heart on Cerebral Diseases.

It has been only within the last twenty years, that the influence of diseases of the heart in inducing apoplexy and other serious disturbances of the brain, has been distinctly appreciated. In the writings of Morgagni, Baglivi, Lieutaud, and even of Corvisart,* we find nothing but merely vague and occasional mention of such a coincidence; and, perhaps, the first who clearly perceived, and unequivocally announced, the connexion between cardiac and encephalic disorders, is Richerand. In the 3d vol. of his Nosographie Chirurgicale, we find the following very expressive sentence:—"The dissection of patients who have died of apoplexy has proved to me, that the excess of force in the left ventricle of the heart is a more powerfully predisposing cause of the disease than a large head and short neck—a state of body which is supposed by most physicians to indicate the apoplectiform conformation;" and, in a memoir which he read before the Ecole de Medecine, he reports, among others, the case of the distinguished philosopher Cabanis, in illustration of the doctrine.

The left ventricle of the heart was found enormously enlarged and greatly thickened. Eight ounces of blood had

* Corvisart states, that he had not quite satisfied himself that he had ever seen a case of apoplexy, which was evidently the effect of cardiac disease, but that the cases reported by Morgagni, Laurent, Lieutaud, Testa, and others, "suffisent pour établir qu'une affection du cœur peut devenir la cause determinante de l'apoplexie."

been effused into the ventricles of the brain, and this effusion had been so violent, that the septum lucidum had been torn through, and the surface of the thalami and corpora striata was rough and jagged. Malpighi and Ramazzini died of similar attacks ; and in their cases also, the heart was greatly enlarged. In 1819, M. Bricheteau communicated a paper to the Journal Complem. des Sciences Medicales, in which he adduced several cases to illustrate the striking influence which hypersarcosis of the heart has in inducing apoplectic seizures. Subsequently to this date, MM. Lallemand, Broussais, Andral, Bouillaud, and others, have directed the attention of the profession to this very important pathological conclusion. M. Rochoux, indeed has both in his treatise on Apoplexy, and in the Dictionnaire de Médecine, art. Apoplexie, attempted to shew, that the coincidence of cephalic with cardiac disease, is by no means so frequent as these authors have, as he says, exaggeratingly announced ; and he refers to the results of forty-two dissections of fatal apoplectic cases, in very few (three only) of which there were any abnormal appearances in the heart discovered. We will not waste time in replying to his reasonings and assertions, as by far the greater number of physicians are quite satisfied of the truth of that remark made by MM. Bertin and Bouillaud, in the Traité des Maladies du Cœur, &c. " that the majority of the patients in whom hypertrophy of the left ventricle is present, will be found to exhibit symptoms of cerebral congestion, and that many of them will fall victims to cerebral disease."

Within the last few years, the connexion of diseases of the heart with those of the brain has been examined by some physicians in reference to the pathology of insanity and other mental disorders. M. Falret of the Hospice de la Salpetriere at Paris, has communicated the results of his dissections in 92 cases of chronic mania. In 20 of these, there were " des lesions diverses du cœur, coincidant avec des alterations chroniques du cerveau, ou des membranes cerebrales." But, as yet, this field of enquiry has not been explored with sufficient care, to entitle us to do more, at present, than to recommend its cultivation to the attentive study of medical men, who devote themselves to the treatment of mental diseases.

M. Bricheteau has narrated twenty-two cases of apoplexy, in all of which hypertrophy of the left ventricle of the heart existed to a greater or less extent. We shall select only one of these. It is interesting for several reasons.

General Foy, distinguished as an able speaker in the French Parliament, had naturally a strong and very vigorous constitution ; his feelings were exceedingly sensitive, and his temper vehement and irascible. From the period of his childhood, he had been subject to palpitations of the heart ; these attacks had, of late, increased in frequency and severity, in consequence of the " travaux du cabinet," previous to taking a part in the " grandes luttes de la tribune." In 1817, the General, being at this time not quite forty years of age, was threatened with an apoplexy. This, as well as some subsequent attacks, were speedily relieved by copious venæsection and other depletory measures, prescribed by the late eminent phrenologist, Dr. Gall. He was afterwards under the professional care of M. Broussais, who had known him in Italy, and who, being aware that his patient had a tendency to heart-disease, regulated his treatment accordingly, with great benefit to his general health.

In 1823, the General experienced an attack of enteritis and nephritis. At this period, the hypertrophy of the heart seemed to have become much aggravated ; and the excessive violence of the pulsations, below the left clavicle, suggested suspicions of the arch of the aorta being diseased. During the following eighteen months, the health of the patient did not experience any decided change : every now and then, indeed, he suffered from the palpitations, cough, and cerebral congestion ; but it was not until the year 1825 that a marked aggravation of his disease took place. Soon after the close of the Parliamentary session of this year, General

Foy fell down quite insensible in the street. Fortunately however this attack was not of long duration. In the course of the Autumn, the celebrated opposition orator travelled to the South; but, unfortunately, he was exposed to numerous causes of excitement, which invariably reproduced his sufferings. The cardiac disease gradually increased, and proved fatal in the following November. The cerebral distress had of late considerably abated ; this was, no doubt, attributable to the state of rest and inactivity, rendered imperative by the palpitations, and other symptoms of thoracic suffering.

*Dissection.* There was effusion to a considerable extent in the cavities of the pleura, and also within the pericardium. The heart was enormously enlarged in volume, and the walls of the left ventricle in particular were much increased in thickness. The head was not examined.

M. Bricheteau sums up his observations, by embodying them in the following six propositions .—

I. The proximity of the heart to the cerebrum (indicated by the comparative length of the neck), and the energy with which the blood is propelled from the one organ to the other, exercise, in health as well as in disease, an influence on the character and " l'étendue" of the cerebral functions, and even of the instinctive and intellectual faculties.

Our author illustrates this position by reference to numerous physiological, as well as pathological phenomena, for most of which he is indebted to the popular writings of M. Richerand. We are reminded, for example, of the old and *well-established* remark, that a short neck and a large head indicate a fertile fancy and a vast intelligence. M. B. admits that numerous exceptions to this law may be adduced; but the truth of it, as a general position, he deems to be indisputable.

That the cerebral circulation is more than usually active during mental exertion, is indicated by the flushing of the countenance, the reddened state of the eyes, the throbbing of the temples, and so forth. The effects of coffee, wine, and spirits in exalting the energies, and in animating the fervour of the mind, at least in many constitutions, are universally known. Most persons can think best in the recumbent position; and some eccentric individuals assure us, that their fancies and memories are most vivid, when they lie with the head lower than the rest of the body.

So much for the arguments which are supposed to prove, that an unusually active state of the cerebral circulation is propitious to intellectual exertion. Let us now see whether the reverse of this condition, viz. that wherein the cerebral circulation is at all opposed and retarded, appears to be correspondingly unpropitious.

M. Bricheteau informs us, that persons who have very long necks, and in whom, consequently, the distance between the heart and the brain is considerable, are not usually endowed " d'une raison superieure, ni d'un esprit brillant ;" but are usually of unimpassioned feelings, and of only moderate intellectual capacities.

The truth of this remark, he thinks, is confirmed by reference to the history of many of the lower animals. Those with long necks, such as the giraffe, the stag, the crane, the goose, and swan, have " un instinct tres borné." Fontaine was too accurate an observer of nature to be guilty of the mistake of ever making these animals hold " un language spirituel," in his fables ; they are usually introduced by him as emblems of stupidity. On the contrary, the elephant, whose immense head is, from the shortness of the neck, brought near to the centre of the circulation, is endowed with a remarkably developed intelligence. The same may be said of the dog, the fox, the bear, beaver, and of many others. Bichat and Legallois have illustrated this curious subject in their writings.

The general inference which M. Bricheteau wishes to establish is, that whenever, from any cause congenital or acquired, the activity of the cerebral circulation is great, the functions of the mind will be found to be correspondingly energetic.

II. Hypertrophy of the left ventricle may induce cerebral congestion, "coups de sang," and even attacks of genuine apoplexy, simply by the abnormal impulsion which is thereby communicated to the circulation within the head; this accident is by no means of rare occurrence.

Indeed, some of the most striking symptoms which indicate the presence of hypertrophy of the heart, are those of cephalic disturbance; the sense of the rushing of the blood to the head on any exertion, or sudden mental excitement, the throbbing, oppressive headaches, aggravated by the recumbent position, the vertigo, the dimness of vision, or the appearance of spots or specks before the eyes, the tinnitus aurium—these are the symptoms which formerly were attributed, on all occasions, to what was called the "apoplectic tendency or disposition of body," but which now are more justly acknowledged to indicate, at least very frequently, the existence of cardiac hypertrophy as their inducing cause. The importance of this pathological doctrine must be too obvious to require any illustration; for surely it must be always desirable to apply our remedies as near to the original seat of the mischief as possible.

III. An over-active impulsion of the blood on the brain may cause either a laceration of the cerebral pulp, or the dilatation and rupture of some of the small vessels, especially when these vessels have become aneurismatic, or otherwise altered in texture—or, lastly, an effusion of the serous part of the blood. Palsies, brain fevers, and various forms of nervous excitability and of mental disturbance, are primarily owing to the excessive impetus of the current of the circulation on the brain, induced by hypertrophy of the heart.

It is the duty, therefore, of the physician, in all such cases, never to omit the examination of the state of the heart and great vessels.

IV. When hypertrophy of the heart [illegible] accompanied with other forms of cardiac disease, especially with such as impede the free egress of the blood from the left cavities; for example, with contraction of the aortic orifice, and ossification of the semilunar valves, the symptoms of cephalic distress are generally much less conspicuous. The dyspnœa, tendency to syncope, and disposition to dropsical effusion, are more marked; but the signs of congestive fulness within the head are less so, than when the hypertrophy is simple and uncomplicated. Even the former train of symptoms is, in some cases, very obscure and indistinct, although the cardiac disease is extensive and considerable, provided there be a correspondence between the hypertrophied state of the ventricle, and the amount of impediment to the free egress of the blood from its cavity, so that the effects of the former are counteracted, and as it were neutralized by the latter.

It is only in this way that we can rationally explain the history of those cases, in which the heart has been found on dissection seriously diseased, although the patient had not indicated, during life, any prominent symptoms of such a lesion. Dr. Hope records a very instructive example of this sort.

An old man, aged 80, died from a surgical complaint in St. George's Hospital. He had never "manifested symptoms of diseased heart sufficient to arrest his own attention, or that of his medical attendants;" and yet, on dissection, "the walls of the left ventricle were found to be at least an inch thick, without any change of its cavity, and the aortic and mitral orifices were respectively encircled by a ring of bone, as thick as a writing-quill."

V. A contracted state of the cavity of the left ventricle favours and accelerates the impulsion towards, and congestion within, the head; and, on the other hand, dilatation of the ventricular cavity has a contrary effect, the heart, though enlarged, being less vigorously contractile under the latter circumstances.

VI. A knowledge of the influence of hypertrophy of the heart on the development of apoplectic and other cerebral diseases, must lead to a more rational and more successful mode of treatment, especially of the preventive or prophylactic treatment.

The occasional application of leeches over the region of the heart, instead of on the temples or any other part of the head, the internal use of digitalis, hydriodate of potass, and other diuretics, and the avoidance of whatever may increase or otherwise disturb the action of the heart, will be found to be the most simple and most decidedly efficacious remedies that can be employed. With respect to the proper place for local bleeding, in such cases as we have been alluding to in this paper, we predict that the practice of repeated cuppings from the nape of the neck will become less common than it has been hitherto. The distressing symptoms indicating congestion of the cerebral symptoms may be mitigated thereby for a time; but the organ, chiefly at fault, is only very partially and indirectly relieved. Even before the correct etiology of numerous cases of apoplexy was understood, the French physicians have been always in the habit of applying leeches to some part at a distance from the head, the anus for example; and probably this practice has been more judicious than that usually followed by English practitioners of applying them on the temples, or behind the ears.—*Clinique Medicale, par M. Bricheteau.*

It will be useful to compare the observations of the most recent authors on diseases of the heart, and of the brain, with the opinions expressed in the preceding remarks of M. Bricheteau. That most indefatigable physician, M. Bouillaud, has in his recent work, reviewed briefly in the present number of this Journal, devoted a few pages to illustrate the influence of hypertrophy of the left ventricle in predisposing to, and in inducing apoplexy and other cerebral disorders.

Out of 54 cases of hypertrophy of the heart (in some of these, the right ventricle only was hypertrophied, and the left one was not at all, or very inconsiderably so) there were eleven, in which cerebral disease, in six apoplexy and in five ramollissement of the substance of the brain, was discovered on dissection. The state of the cerebral arteries is indicated in seven only of these cases;—in four they were ossified and cretaceous at some point, or points; in two, they were perfectly sound; and in the remaining one, their walls were only "un peu blanches, et epaissies." In six of the eleven cases, the hypertrophy of the left ventricle was excentric, or accompanied with dilatation, (the active aneurism of Corvisart); in three, it was concentric, or accompanied with contraction of its cavity; and in two, the hypertrophy was simple, the cavity remaining of its normal dimensions. Allusion is made to the case published many years ago by Legallois in the Bulletin de la Faculté, and entitled, "Observation sur une maladie du cœur par hypersarcose, ou par exces de la substance musculaire de ce viscere, cause presumée d'une apoplexie foudroyante." The case occurred in a female, 25 years of age, who had laboured under symptoms of heart-disease from her infancy, and who had been subject to severe head-aches, and occasional bleedings from the nose. She died of a sudden apoplectic seizure. On dissection, the walls of the aortic ventricle were found extraordinarily thickened and indurated, but its cavity was not larger than in health. In the right ventricle of the brain, there were at least three ounces of coagulated blood; the septum lucidum was partially torn, and some of the blood had apparently escaped through the rupture into the left ventricle.

In reference to the adverse sentiments of M. Rochoux, (who maintains that cerebral hæmorrhage is almost invariably preceded by, and dependent on, a peculiar softening of a certain portion of the substance of the brain, *ramollissement hemorrhagipare,* and is very rarely associated with cardiac disease,) M. Bouillaud very judiciously says, that it is only by accurate statistical tables of disease, that we can hope to determine the point at issue.

He admits that in numerous cases of apoplexy, complicated with heart-disease, especially with hypertrophy of the left ventricle, the cerebral arteries are at the same time cretaceous and fragile; and also that cerebral hæmorrhage is not unfrequently discovered in persons who have not been affected with any disease of the heart; but at the same time he contends, that the morbid alteration of the cerebral arteries is by no means of constant and uniform occurrence, and that the coexistence of an hypertrophied state of the left ventricle with cerebral hæmorrhage is sufficiently frequent to warrant us in regarding these diseases as related to each other, in the light of cause and effect.

Dr. Copland announces similar opinions in his Medical Dictionary: "The frequency," says he, "of apoplexy, or palsy in the advanced progress of cardiac disease, especially hypertrophy of the left ventricle, have led me to believe that more than mere coincidence actually exists;" and with great discrimination he adds, "It is however by no means improbable, that the disposition to organic change throughout the whole vascular system, sometimes associated with disease of the heart, may so far exist in the delicate vessels of the brain, as to favour the occurrence of hæmorrhage from them, when the action or impulse of the heart is increased by disease, or the influence of passion, or emotion; or when the return of blood from the head is impeded by congestion, or interrupted circulation through the lungs, or right side of the heart."

We cannot but express our surprise at the almost total omission of any reference to the connexion between cerebral diseases and those of the heart, in the article on apoplexy communicated by Dr. Clutterbuck to the Cyclopædia of Practical Medicine, a work which, although containing some valuable medical essays, is very imperfect and incomplete. The only remark made on this most *practical* subject is, where he enumerates among the causes which operate by impeding the return of blood from the brain, "diseases of the heart or lungs, impeding the transmission of the blood through the pulmonary vessels."

Dr. Hope read last year before the College of Physicians a paper on the connexion of apoplexy and palsy with organic disease of the heart. Out of 39 fatal cases of apoplexy, disease of the heart was found co-existent in 28. According to his data, the author concludes that the periods of life at which fatal apoplexy is most prevalent, are precisely those in which disease of the heart (either hypertrophy of the muscular substance or ossification of the valves and vessels) is of most frequent occurrence—namely, between forty and fifty, and between seventy and eighty. The practical inferences which Dr. Hope derives from his enquiries are simple and judicious. He particularly insists upon the necessity of avoiding all severe bodily exercise, and all exciting emotions of the mind. The paramount object in this, as in every other disease, is a correct diagnosis. The appropriate treatment follows almost of course.*

---

* The two most recent French and English authors on diseases of the heart, it appears to us, are very unwilling to do justice to each other. They have both been playing too much of a see-sawing game of borrowing and self-appropriation, without due acknowledgment of each other's merits and discoveries. In the article hypertrophy of the heart, in the Cyclopædia of Practical Medicine, we observe that more than one passage has been literally translated from an old article in the Archives Générales, and from Bertin's treatise, no reference however being made to either of these works; and on the other hand, Bouillaud, in his "Traité des Maladies du Cœur," published but last year, very gravely tells us that, at the time he performed some experiments in

Many most interesting cases illustrative of the connexion, or at least of the co-existence of the cardiac and cephalic disease have been published in the pages of this Journal. We may refer in particular to one which occurred in the practice of Dr. Seymour at St. George's Hospital, narrated in the 11th vol. The patient died apoplectic. A large sanguineous clot was found in the right ventricle of the brain; the basilar arteries exhibited spots of ossification; the walls of the left ventricle of the heart were greatly thickened; and the semilunar valves of the aorta and also the coronary arteries were rigid and hard at several points.

In the same volume, the Senior Editor of this Journal has narrated at great length a most instructive case of hypertrophy, &c. of the heart, where, although the cardiac disease was probably the primary evil, and the cause of the cerebral affection, it had been almost totally overlooked.

---

### On the Influence of Lesions of the Right Ventricle of the Heart on the Pulmonary Circulation.

We have shewn in the preceding article that many of the lesions of the brain are directly owing to a previous disturbance of the functions of the heart; this disturbance consisting in an over-activity and strength of the left ventricle, induced by hypertrophy or, as some prefer to call it, hypersarcosis of its muscular parietes. We shall now briefly consider the reciprocal influences of diseases of the right ventricle on the state of the lungs, and of diseases of the lungs on the state of the right cavities of the heart. Some physicians of the last century had alluded to these pathological relations; but they had done so only in a very cursory manner; and it was not until Laennec in the first instance, and subsequently Bertin, Bouillaud and others, following the path of their great leader, had analysed and arranged the two important departments of pathology, which treat of the diseases of heart and of the lungs, that the intimate connexion of these vital organs in their morbid states, and the reciprocal influences of the one in inducing disorders of the other, was distinctly stated and satisfactorily unfolded. True it is, that since the days of Hippocrates, it has been well known that diseases of the heart almost invariably induce serous effusions into the texture of the lungs, and into the cavities of the pleuræ; and, on the other hand, that one of the most constant of the symptoms of hydrothorax is violent and irregular palpitation of the heart; but physicians had not, until within the last five years, been in the habit of watching the steps or gradations, by which the diseases of the one organ operate in inducing those of the other: the general facts were known, but known only at a period of the diseases, at which the knowledge could be comparatively of little use in suggesting appropriate remedies for their relief. It is only during the earlier stages of pulmonary and cardiac diseases that we can reasonably hope to cure, or mitigate them; and great and most gratifying credit the scientific physician may acquire by his skilful treatment. For this purpose he must endeavour to ascertain which is the real "fons et origo mali," and which is the secondary or the superinduced disease. The extreme frequency of heart disease, (a frequency which, it almost seems, has greatly increased of late years,) will suggest the propriety of treatment directed to the region of the heart, in many cases of apparently idiopathic pulmonary disease; and the simple remedy of occasionally applying three or four leeches to this part will often produce the most pleasing effects.

---

to disprove the accuracy of Majendie's theory on the sounds of the heart, he was not at all aware that Dr. Hope had anticipated him in a similar enquiry, although the English work had been published at least two years previously, and was perfectly well known on the Continent.

All this is not worthy of the generosity of science.

M. Begin, in a very valable paper on asthma, in the Journ. Compl. des Sciences Medicales, has very forcibly stated the mutual relations between the lungs and heart in this disease. "In order that the lungs may perform their functions healthily, they must afford a free and easy passage to the blood circulating through them. Whenever the due expansion of the lungs is from any cause impeded, the blood accumulated in the right cavities of the heart must overload them, and their muscular parietes being excited to unusual efforts to expel their contents will, in course of time, become the seat of organic changes. The obstructed state of the lungs is the first link in the chain, and is in truth the morbific cause of the cardiac lesions. It is by inducing primarily a congested state of the pulmonary circulation that all over violent or protracted exertions of the voice, as in practising on wind-instruments, and all unusually severe muscular efforts, are so apt to lay the foundation of heart disease.

When a portion of one or of both lungs is irremediably hepatised, or when a pleuritic effusion exists to such an amount as to compress the lungs, the pulmonary circulation must be more or less embarrassed; and we cannot, therefore, be surprised that the heart may eventually suffer some organic change. The repeated recurrence of paroxysms of asthma must necessarily have a similar effect."

If we admit that an obstruction to the free transmission of the blood through the lungs causes a regurgitation back on the right cavities of the heart, and is thus apt to induce a dilatation of these cavities, it is no less probable that the action of the right ventricle, when it is primarily hypertrophied, may induce pulmonary congestions, hæmoptysis, and suffocation.

Many cases in truth of these forms of disease are explicable in the way we have now noticed. The heart is primarily the offending organ, and the practice therefore of a judicious physician ought to be directed, in an especial manner, to its relief. The course of the pulmonary artery is so short, that the impulsion of the current from the right ventricle is communicated directly, and with no abatement of its energy, on the structure of the lungs. If, therefore, from any cause, as from hereditary predisposition, or from any antecedent disease, the textures of the lungs are unusually delicate and lacerable, we may easily understand how hypertrophy of the right ventricle will be apt to induce hæmoptysis, or pulmonary apoplexy. The influence of the right ventricle on the lungs must be greater than that of the left ventricle on the brain. In the one case, there is no impediment or obstruction to the impetus of the blood; while in the other it must be more or less abated by the long and tortuous course of the carotid and vertebral arteries, and by the minute subdivision of these vessels, before they are distributed on the encephalon.

M. Bricheteau has made several experiments on the dead body, which prove how liable the minute pulmonary arteries must be to rupture from any unusual impetus of the circulation. He has often found that by throwing in an injection into the pulmonary artery with very moderate force, the vessels have given way at one or more points of the lungs, and the parenchyma has been infiltrated with the injected matter.

These considerations lead us to appreciate with accuracy the rationale of the symptoms which are induced by hypertrophy of the right ventricle of the heart.

The palpitations are always much aggravated by whatever distresses the breathing, and impedes the free circulation of the blood through the pulmonary vessels; the pulse at the wrist is not necessarily much affected, at least not in the same degree, as we find it to be usually in hypertrophy of the left ventricle; the face and lips have a purplish or livid hue; the jugular veins are full, prominent, and sometimes pulsatory; the respiration is short and much harassed on any exertion or agitation; the blood is often imperfectly oxygenated, and when this is the case, the functions of the brain are sluggish, and the patient, towards the close of the disease, becomes lethargic, or even quite comatose. Dropsical effusions are of more frequent occurrence from hypertrophy of the right, than of the left ventricle.

M. Bricheteau alludes particularly to the occurrence of repeated hæmoptysis (when this is not symptomatic of tubercles), as one of the least fallible signs of hypertrophy of the right ventricle. When spitting of blood occurs for the first time in persons of middle or of advanced years, and especially if it is accompanied with palpitations, and other symptoms of cardiac distress, we have strong reason to suspect that the right ventricle is hypertrophied, and that the effusion of blood is the direct consequence of this morbid state.

M. B. has published reports of six cases, which illustrate this connexion between hypertrophy of the right ventricle and some sorts of hæmoptysis. Two of the cases were fatal, and dissection confirmed the accuracy of the diagnosis. The ages of the patients varied from 26 to 58; no mention is made that any of them had a phthisical tendency. We shall present to our readers abbreviated reports of two of the cases, selecting a fatal and a fortunate one.

*Case* 1. A groom, 48 years of age, plethoric and robust, became subject six years ago, after a severe attack of pneumonia, to frequent palpitations of the heart. His breathing became gradually more and more oppressed, especially on any exertion, and, above all, whenever he attempted to mount up stairs. From time to time he expectorated a quantity of blood. Œdema of the limbs had of late supervened.

When admitted into the Hospital Necker, of which M. Bricheteau is one of the physicians, the symptoms of cardiac and pulmonary disease were strongly marked. The face was purplish and swollen; the jugular veins were distended, and pulsated regularly like an artery; the breathing was short and quick; the heart throbbed violently, especially on its right half; and a frequent cough, accompanied with copious expectoration, harassed the patient; the pulse at the wrist was rapid, hard, and irregular. At almost every point of the chest, a strong respiratory murmur, blended with a loud mucous râle, was audible. The patient was obliged to keep constantly the sitting position. In a few days he died, quite asphyxiated.

*Dissection.* There were two ounces of serum within the pericardium; the left cavities of the heart were normal—the right cavities were much dilated and thickened. The left lung was hepatized to a great extent, and here and there exhibited spots of half-coagulated blood.

*Case* 2. A woman, 40 years of age, of a healthy and sanguineous temperament was seized, after some laborious occupation, with a copious hæmoptysis. This attack was speedily checked; but, in the course of a few days, the spitting of blood returned. It continued for two days. No abnormal respiratory sound could be detected by auscultation; but the pulsations of the heart were extended over a larger space than is usual; they were irregular and intermittent, and were strongest over the right side. The jugular veins near the sternum exhibited regular pulsatory movements. The pulse at the wrist was but little affected in any respect. Venæsection, and the use of cooling demulcents arrested the hæmorrhage; but there were several subsequent relapses, in consequence of the patient exerting herself. At no time did auscultation reveal any abnormal respiratory sounds.

The other four cases reported by M. Bricheteau agree with the preceding in the general features, of the hæmoptysis being attended with symptoms, more or less distinctly marked, of disease of the right side of the heart, and of its being quite independent of any tubercular ulceration. The importance of discriminating these two forms of hæmoptysis, the one from the other, must be obvious to every one. The age and constitution of the patient, the presence or absence of tubercular disease on the one hand, and of cardiac disease on the other, ought to be accurately investigated; and, from the improved means of diagnosis now adopted, we may generally determine the genuine nature of the case.—*Clinique Médicale, par M. Bricheteau.*

M. Bouillaud, in his excellent treatise on the Diseases of the Heart, states that he has met with only three cases of pulmonary apoplexy or hæmorrhage, in patients affected with hypertrophy of the right ventricle of the heart.

He admits that this number is far too limited to warrant any general conclusions; but he assures us that he is quite satisfied that these two morbid states are connected with each other, in the relation of cause and effect. That this idea is one not of recent adoption on his part, he refers to an article published by him so far back as the year 1824, in the Archives Génerales, where it is stated :—

"When the walls of the right ventricle are increased in thickness and strength, they must communicate to the pulmonary circulation an activity and force proportionate to their hypertrophy, and sufficient sometimes to overcome the resistance of the vessels; hence the origin of pulmonary hæmorrhages, or apoplexies (coups de sang), which are induced in a manner quite similar to that in which cerebral apoplexies are so frequently caused by hypertrophy of the left ventricle."

M. Bouillaud admits that pulmonary apoplexy from hypertrophy of the right ventricle, is by no means of such frequent occurrence as cerebral apoplexy from hypertrophy of the left one. There are two good reasons for this; in the first place, hypertrophy of the right ventricle is not nearly so common as hypertrophy of the left one; and, secondly, the pulmonary arteries are very rarely affected with that calcareous degeneration, which is so frequently observed affecting the branches of the internal carotid.

M. Chomel, in the Dictionnaire de Médécine, art. Hemoptysie, alludes to the occasional dependence of the disease on hypertrophy of the right ventricle; but he adds that this is of extremely rare occurrence, and that by far the most frequent cause of pulmonary hæmorrhage is tubercular ulceration.

"True it is (says he), that it may be sometimes induced by violent efforts or strainings, from congestion of the pulmonary veins, occasioned by thoracic or even by abdominal disease; and now and then it appears to be a salutary effort of Nature to relieve herself, as we witness in some cases of amenorrhœa; but for one case arising in any of these ways, there are at least a dozen or twenty which can be traced to phthisical degeneration of the pulmonary texture."

It is, however, to be remembered that M. Chomel has, in the article alluded to, evidently confined his remarks to that form of hæmoptysis which is so generally associated with tubercular disease; this form, or the phthisical hæmoptysis, is generally (but certainly not always) comparatively slow and gradual, whereas that connected with disease of the heart is copious and sudden, and is well denoted by the phrase "coup de sang," which the French authors often use.

In the valuable article on pulmonary apoplexy, communicated by Dr. Townshend to the Cyclopædia of Practical Medicine, we find the best summary of authentic data, illustrative of the connexion between this malady and disease of the heart. He says :—"Of twenty cases of pulmonary apoplexy that we have examined after death, fifteen, or rather more than two-thirds of the whole, occurred in individuals labouring under heart disease; in two instances only was the pulmonary apoplexy connected with tubercles in the lungs; in one, the disease was caused by external injury; and in four cases we could not trace the hæmorrhage to any pre-existing disease, either of the heart or lungs."

The late Mr. Burns, of Glasgow, has pointed out the connexion of pulmonary hæmorrhage with some diseases of the heart in very forcible language :—"The pulmonic vessels, by the congestion and continued vis à tergo, are ruptured—blood forced into the air-cells, or, if urged still further, all the cellular structure of the lungs is crammed with blood; these organs cut like liver, and sink when put into water." No description of what in the present day is called pulmonary apoplexy, could be better.

The lesion of the heart, with which this disease is almost always connected, is an hypertrophied state of the right ventricle. That the simple contraction of the left auriculo-ventricular orifice, the right ventricle being normal, may by causing an obstruction to the free return of blood through the pulmonary veins, occasion a rupture of the pulmonary capillaries, is admitted by all authors who have of late written on the subject; but we are assured by Bertin and others, that pulmonary hæmorrhage from this cause is not only much less common, but also always more gradual in its occurrence, and more passive in its character, than that which is produced by hypertrophy of the right ventricle. The following case, related by Dr. Law in the Cyclopædia, affords a good example of the co-existence of both lesions.

"A young lady had, for three years, laboured under most distressing palpitation, greatly increased by the slightest exertion or motion; auscultation left no doubt that the lesion consisted in a narrowing of the left auriculo-ventricular orifice. She had repeated hæmoptysis, which became more frequent and profuse as she approached death.

On dissection, both lungs were found gorged with blood; even the minutest capillaries under the pleura were injected, and there were numerous ecchymosed patches in the subjacent cellular tissue. The right ventricle of the heart was hypertrophied and dilated—the left ventricle was smaller than natural—the left auricle was dilated and thickened, and the left auriculo-ventricular orifice was so contracted, that it would scarcely admit the introduction of a crow-quill! The pulmonary artery was larger than usual, and the aorta was very considerably smaller." The remark which Dr. Law appends to this report, viz. "that this (the narrowing of the left auriculo-ventricular orifice) is the lesion which we shall find to be most frequently the cause of pulmonary apoplexy," is at variance with the testimony of Bertin, Bouillaud, Townshend, and others, and, indeed, with Dr. Law himself, who rather inconsistently observes, in a subsequent part of his own paper—"pulmonary apoplexy is found to occur most commonly from hypertrophy of the right ventricle."

Such are some of the most authentic data which illustrate the position which we proposed to establish, that one form of hæmoptysis is very generally connected with, and in truth dependent upon, hypertrophy of the right ventricle.

---

### Memoirs of the French Royal Academy of Medicine.

The last published fasciculus of these Memoirs contains very few papers of general interest. The learned Society must surely be at a great loss for valuable communications, when more than three-fourths of a fasciculus are occupied with such articles as an "Eloge de Chaussier," wire-drawn to the portentous extent of forty quarto pages—a "Notice sur la Peste de Moscow," which prevailed upwards of sixty years ago; and an account of the changes effected in the treatment of the insane at the Bicêtre by the late M. Pinel, so far back as the year 1792, and copied from some manuscript papers, which were probably never intended for publication. Besides these uninteresting papers there is another, on that variety of inguinal hernia, in which the displaced gut does not protrude through the external inguinal aperture, being confined in the passage between this and the internal aperture, and which has been denominated the incomplete inguinal, the intra-inguinal, or the inguino-interstitial hernia. There is no new matter in this essay from the pen of Dr. Goyraud, and it need not, therefore, occupy our attention. The remaining two papers are somewhat more deserving of notice, although, in our opinion, far from being worthy of the Transactions of the Royal Academy of Paris. Their sum and substance are contained in the following analyses.

I. On the Introduction of Air into the Cavities of Bloodvessels:—On the Torsion of Arteries as a Means of arresting Hæmorrhage:—Surgical Report of M. Clot Bey.

M. Amussat has had lately several opportunities of witnessing the occurrence of this fatal accident—the introduction of air into the cavities of bloodvessels, even when the size of the wound was very small. He urges, therefore, the necessity of extreme caution, whenever the jugular, or other veins adjacent to the heart are opened or divided, and recommends that compression below the wound should be steadily kept up, as long as the blood continues to flow. The numerous experiments which he has performed on the lower animals, for the purpose of elucidating this, and various other points connected with traumatic hæmorrhages, have proved to him, that the most effectual remedy consists in immediately closing the opening of the vein (through which the air has rushed in with a peculiar whizzing or bubbling sound), and in compressing rapidly, and "par saccade," the thorax and abdomen, the finger being placed on the wound during each interval of compression. By these means, M. Amussat has succeeded in saving many animals, in which air had been introduced into the heart from a wounded vein, either accidentally or by design; whereas those animals (he says) which were left unassisted invariably perished. In addition to the means now mentioned, we may try to suck out the absorbed air by means of a tube, either with the mouth, or, as Majendie proposes, with a syringe. Immediately afterwards, the wounded vein should be secured by ligature or by torsion.

There are two principal methods employed in the present day to arrest alarming hæmorrhages—the ligature and torsion. Hitherto, the latter method has not met with all the encouragement which its simplicity and success deserve. It has, however, been making a steady, although it may be a slow progress, and the reader will find by some of the following details that several of the best French surgeons are in the habit of adopting it.

The complete isolation of an artery is not an indispensable preliminary to the application of a ligature; but it is so to the employment of torsion. A very simple manœuvre, however, with two forceps, one held in either hand, will easily attain this object. There is more difficulty in exposing properly the arteries of the scalp, than those of any other part, in consequence of their firm connexion with the close and indurated cellular tissue there. But this difficulty is quite counterbalanced, by the less risk of erysipelatous inflammation after torsion, than after the application of a ligature to these vessels.

As soon as the vessel is properly isolated, or detached from its adhesions, the operation of torsion is half completed; whereas the use of a ligature very generally requires the aid of an assistant. In the present state of the science, torsion is, we must confess, a more delicate and difficult operation than the ligature; but a few trials of it on living animals will speedily enable any surgeon to adopt it with perfect facility. From the time when I first devised torsion, says M. Amussat, I have not applied a single ligature; I have employed it in all, even in the most important operations of surgery, with the utmost facility and with perfect success." The advantages of torsion over the use of the ligature are the following:

The operator is independent of any assistance; the artery is "sufficient for itself," and no extraneous object is introduced into the wound; and, lastly, the danger of consecutive hæmorrhage is, in M. Amussat's opinion, less. He quotes from the French Lancet of Jan. 1834, a case reported by Dr. Ferrus, surgeon of the Military Hospital at Calvi, in Corsica, where the patient might have been lost in consequence of the unskilfulness of the assistant, had Dr. F. not immediately had recourse to the torsion of the bleeding vessels.

M. Clot-Bey has employed torsion after several operations. In a surgical report of his hospital at Abou Zabel in Egypt, published recently in the Journal Hebdomadaire, we find a notice of an amputation of the arm, where he had recourse to torsion with perfect success. After laying hold of the bleeding vessels with the forceps, he twisted them round four or five times, without any previous "refoulement" of their inner coats or membranes. The success which attends all the operations of the Bey is indeed quite marvellous. With becoming modesty he attributes it to the favorable constitutions of his patients, and to the salubrity of the climate of Egypt. A case of disease of the lower jaw is mentioned, in which one half of the bone was extirpated: the cure was perfect by the 12th day after the operation. Caries of the ribs and sternum seems to be of more frequent occurrence in Egypt than with us. The following two cases may deserve notice. A young Arab soldier had for some time felt a pain in a limited spot below the left clavicle. He attributed it to a blow he had received from one of his comrades. A swelling had formed at the part, and had become gradually larger, till it was of the size of an egg at the period of his admission into the hospital. An incision being made into it, a quantity of pus was discharged, and the bone was found to be exposed and carious for the space of an inch. M. Clot determined to excise the diseased portion of rib. The intercostal muscles having been divided, and the pleura detached by means of a blunt spatula, the cartilaginous end of the rib was cut through by means of a very strong scalpel and elevated; a chain saw was then passed under the rib, and a portion of it, an inch and a half long, was separated. The intercostal artery was tied, and some charpie placed in the wound. The cure was speedily complete.

The second case also occurred in an Arab soldier. A tumor had very gradually formed on the posterior and lower part of the left side of the thorax. A little higher up than the tumor itself, there was a small aperture. A probe being introduced, the rib was found to be carious. On laying open the tumor, upwards of half a pint of unhealthy pus was discharged. The intercostal muscles were then cautiously divided, and the pleura separated; a chain-saw was passed behind the diseased ribs, and portions of the seventh and eighth ribs, the one two and the other three inches in length, were sawed off. The lips of the wound were retained together by means of a twisted suture. For the first four days after the operation, there was much febrile irritation, but this gradually subsided, and the patient eventually recovered his health entirely.

M. Clot alludes to two cases of elephantiasis of the labia pudendi in young Arab females. In the first case, the enlargement being not very considerable, the treatment was limited to the application of leeches, and of hydriodated ointment; but in the second, the right labium had become so enormously enlarged, that the patient willingly submitted to its excision. The operation was quickly done, and with perfect success.

In neither case could M. Clot ascertain a probable cause of this curious malady.

To return from this digression on M. Clot's surgical clinic to the subject of the torsion of arteries, the following memoranda of cases, in which M. Blandin employed it at the Hospital Beaujon, and published in the Journal Hebdomadaire, may be deemed instructive.

After the excision of a scirrhous mamma, the bleeding vessels were successively seized with the forceps and twisted. some of them could not be sufficiently isolated, and others tore or escaped from the hold of the forceps several times, before the hæmorrhage was completely arrested. On the second day after the operation, the wound was dressed: the lips were tender and swollen, and an erysipelatous redness was diffused all round; several small clots of blood escaped from the wound. On the following day the poor woman died very unexpectedly. On dissection, the lips of the wound were found partially united, and lifted up "en totalité" by a quantity of coagulated fetid blood underneath. The subcutaneous cellular tissue was infiltrated with purulent matter.

(This case is certainly not very favorable to torsion: perhaps it was not cleverly executed.—*Rev.*)

In the second case, the torsion succeeded better.

A young girl had her leg amputated. "The anterior and posterior tibial, and the peroneal arteries were successively seized with the torsion-forceps, drawn out from their surrounding attachments, laid hold of and squeezed between the branches of a second forceps, so that the internal and middle tunics might be lacerated, then drawn between the branches of this forceps, so that these membranes might be folded or rolled back, 'refoulé,' and lastly, the cellular or outer membrane only was twisted, the vessel being kept firm all the while by means of the second forceps."

(This description is certainly not very perspicuous.)

The hæmorrhage was quite arrested from the three vessels, and their pulsating ends were distinctly seen on the surface of the wound. Cicatrization appears to have proceeded very slowly in this case, as the wound was not entirely healed for nearly three months. At no time, however, was there any threatening of hæmorrhage.

In an amputation of the thigh of a young boy, M. Blandin again had recourse to torsion. "The femoral artery was seized, isolated, its internal and middle membranes ruptured, and "refoulées" between the two branches of the second forceps, and the outer or cellular membrane twisted, while the vessel was kept steady by means of the second forceps. The deep femoral artery was treated in the same manner." Several of the smaller vessels were seized and twisted, but without inducing any laceration or "refoulement" of their inner coats, being simply held between the thumb and fore-finger of the left hand, while the torsion was effected with the forceps in the right hand. The healing of the wound was very slow, but ultimately complete. No hæmorrhage had occurred at any time.

The fourth was a case of excision of the mamma.

Several arteries were successively twisted, in some with, and in others without previous rupture and "refoulement" of their inner coats. The hæmorrhage from all was effectually arrested: the ends of the vessels were seen pulsating at different points of the wound.

The fifth was a case of extirpation of the testicle. The sixth was one of amputation of the thigh in an adult. The femoral and other arteries had been secured by torsion. The patient having died ten days after the operation, these vessels were carefully examined. The femoral artery, where the profunda was given off immediately below the femoral arch, contained a filiform coagulum, which extended to its divided end on the face of the stump, and became much larger near this point, occupying here the entire tube of the vessel. The coagulum adhered firmly to the circular groove, which had been made by the second forceps compressing tightly the artery. The inner and middle coats had been divided at this point, and rolled back, "refoulées," for the extent of a line or two. The twisted end of the outer coat was softened, suppurating, incompletely detached, but separated by gentle traction. The tube of the artery was contracted, and its inner surface exhibited numerous transverse folds.

The next case was one also of amputation of the thigh. The patient died on the ninth day from hectic irritation, but without any hæmorrhage. The femoral artery, as high up as the giving off of the profunda, was contracted, and exhibited numerous tranverse folds.

An inch above its divided extremity there was a small fibrinous clot, of a pale red colour, adhering firmly to one part of the tube, but not filling it up entirely. Below the coagulum, the artery was quite empty. The gaping cellular tunic was in a state of suppuration, and was not to be easily distinguished from the rest of the stump. The internal coats were "refoulées" to a small extent and suppurating. Four other minor cases are related, in all of which torsion was successfully employed to arrest arterial hæmorrhage. The catalogue, therefore, of cases is sufficiently large to demonstrate the safety and efficacy of the operation. In one case only, the first, there appeared to be a want of success; and this was attributable perhaps to the circumstance of the operator being not familiar at the time with the best mode of performing the torsion. Besides, it is not at all improbable that the hæmorrhage may have proceeded from some of the small vessels, which began to ooze out blood, after the wound had been closed. The success of the practice in all the other cases was complete. We cannot, therefore, place much credit on the reports of those surgeons who have announced that torsion has frequently failed in their practice. It is quite possible that the failure has been owing rather to an error of the operator, than to any defect of the operation. The torsion of the larger arteries requires a rather more complex manipulation than when the smaller ones only are to be secured. The use of the two forceps, the seizure and isolation of the vessel with the one, the division and "refoulement" of the inner coats with the other, and, lastly, the torsion of the cellular coat only with the former, the vessel being all the while kept steady with the latter forceps—these different steps must be attended to in the torsion of the larger arteries. The smaller ones are more easily managed: we have only to seize the bleeding end with the forceps, draw it gently out, and isolate it from its connexions, lay hold of it with the thumb and fore-finger of the left hand as high up as we can, and then twist it with the forceps in the right, without any preliminary division and "refoulement" of its inner coats.

The admirable researches of M. Amussat have shown, that the consequence of the "refoulement" of the inner coats of the artery is the exudation of plastic lymph, which agglutinates the sides to each other, and to the extremity of the inclosed coagulum.

In one of the cases which we have related from M. Blandin's practice, we have seen that the fibrinous coagulum adhered firmly to the extremity of the valvula formed by the "refoulement" of the inner membranes of the vessel.

### II. On the Preservation of Timber from the Dry Rot by the Corrosive Sublimate of Mercury.

The Minister for the Marine and Colonies of France applied a short time ago to the Royal Academy for their opinion respecting the efficacy of a solution of corrosive sublimate as a preservative of the timber, sails, and cordage of ships. A commission of five members was appointed to make the requisite inquiries, and to draw up a report of their proceedings. The report commences with a rapid survey of the various means which had been proposed and tried previous to the introduction of the corrosive sublimate. Among the numerous preservatives at different times recommended, we may enumerate various resins, animal, vegetable, and mineral oils, the muriate of soda, the nitrate of potass, quick-lime, baryta, the species of pyrites (?), which the English call "mundic," and which is partly composed of arsenic. This latter substance mixed with water was used to wash the timbers of the Queen Charlotte with; but the shipwrights employed in the work suffered severely from swellings of the glands, and two of them died in consequence. A practice which has been for a great length of time in use in many arsenals is to keep all their store or reserve timber floating in or sunk under sea-water: but this method too has its disadvantages; for the wood thereby loses its proper density and strength, and it cannot, but with difficulty, be afterwards ever thoroughly dried. The saline matter, with which it has become impregnated, is apt to dissolve whenever the atmosphere is damp; and thus a vessel built of such timber is inevitably rendered uncomfortable and even unhealthy. The oxidation too of the iron and copper work is greatly accelerated by the presence of the muriate of soda. Lastly, it may be stated that the wood thus seasoned is by no means exempt from the very evil which the remedy has been used to counteract. In order that timber may remain sound for a length of time, it is necessary that it be thoroughly dried. The drying of timber is usually effected by exposing it for a length of time (under shelter if possible) to the air. But this being a tedious process, (three years may be stated as the average time required,) recourse has been had to artificial warmth. A too high degree of heat weakens the ligneous fibre, and by dissipating all its humidity—a certain degree of which is necessary to its suppleness and strength—renders it friable and disposed to split. It has been recommended to dry and to preserve timber in beds of dried sand; but this plan is evidently inapplicable for large quantities of wood.

The process of that disorganization of wood, so well known by the name of the dry-rot, has been repeatedly examined. The following appear to be the progressive steps of this destructive change. The wood being more or less humid, whether from retaining part of its natural humidity, or, in consequence of the long immersion to which it was during its seasoning subjected, commences to undergo a sort of heating or fermentation, the result of some internal molecular movement, analogous to that which takes place during the decomposition of animal matters. By this fermentation and dissolution of the juices of the wood, the reticular ligneous tissue gradually softens and cracks; and in the cracks or fissures thus formed various cryptogamic plants begin to vegetate. The vegetations, which are in truth not the primary causes of the mischief, but are rather the result of the preceding changes, multiply and increase in every direction, breaking up and disorganizing the ligneous tissue. This development of regular organized vegetable productions succeeding to a previous decay of the wood, may be aptly compared to the generation of worms in putrefying animal matter.

Such appears to be the process of what has been called the dry-rot, or caries of wood; a morbid action which commences with a sort of intestine fermentation or decomposition of the ligneous fibre, and terminates by the production of various cryptogamic plants in its tissue. It had been long well known that the corrosive sublimate of mercury has a powerful effect in arresting the putrid fermentation and decomposition of animal matters. It had been used in preserving antimonial preparations, objects of natural history, in embalming, &c. The most putrescent structures, such as the nervous pulp, acquires a firmness from the sublimate, which enables it to resist decay. Botanists, too, had frequently employed it in the preservation of their herbaria. So far back as the year 1815, Dr. Bailli of Toulouse had suggested the expedient of immersing wood in a solution of corrosive sublimate to preserve it from speedy decay. But we do not propose to dispute the claims of the Englishman, Mr. Kyan, who has recently brought the proposal more formally and more minutely under the attention of his own as well as of our Government.

The solution which he recommends is made by dissolving about a pound of the salt in about eight or ten gallons of water. The requisite period for maceration must vary, according to thickness and hardness of the timber, from a week to three or more. The sails and cordage do not require more than a couple of days.

Several experiments were made at Woolwich to ascertain the preservative powers of Mr. Kyan's solution. Pieces of wood which had been subjected to it, and others which had not, were left for a twelvemonth in a ditch. A quantity of decayed and rotting timber had been thrown in along with the sound, and the whole had been kept rather warm, for the purpose of encouraging the fermentation.

When removed at the end of the twelvemonth, the *prepared* wood was found to be perfectly sound throughout, while the other pieces were all more or less rotten.

The French chemist, M. Henry, has made numerous experiments to ascertain the mode, in which the sublimate probably operates, as a preservative of timber. He has shewn that the salt so employed is in a great measure speedily converted into the state of protochloride, and that, in passing to this state, it forms a combination with the albuminous juices of the wood. There is thus formed a new organic compound, fixed and insoluble, and the vegetable juices are no longer liable to be affected by moisture, or susceptible of that fermentation, which we regard to be the primary and essential stage of the dry rot.

In reference to the question whether the wood, &c. prepared by Mr. Kyan's method is likely to prove hurtful to the shipwrights, or to the crew of a ship which has been built of it, we may allude to some observations of Mr. Faraday. This distinguished chemist informs us that many years ago, his great predecessor, Sir H. Davy, was consulted by the late Lord Spencer, as to the propriety of using the corrosive sublimate to preserve his valuable library from the attack of insects. Both Sir H. and Mr. Faraday were rather unfriendly to its use, on the ground that it might become volatilised, and thus impregnate the atmosphere of the rooms with poisonous particles. But Mr. F. has subsequently changed his opinion, and he is now convinced that his fears were unnecessary. As a matter of course there is infinitely less risk of any such injurious consequences from wood which has been merely saturated in a solution of the salt, than from using the powder of the salt itself to sprinkle on books, or other objects. But we have already stated that the prepared wood retains very little of the undecomposed poisonous salt, the greater part of it being speedily converted into the innocent proto-chloride. The efflorescence which may be often observed on wood which has been immersed in the preserving liquor, consists chiefly of this latter compound.

Already an experiment has been made in England on a large scale, to ascertain whether there is any truth in the objections which have been urged against Mr. Kyan's proposal. A South Sea Whaling vessel was built some time ago at Cowes, altogether of timber which had been seasoned as Mr. K. recommends. No accident occurred among the workmen in the building-yard, and the crew had lived for two months on board of the ship before she sailed, without experiencing any inconveniencies. It will be interesting to know the result of the experiment when she returns after a two, or three years' voyage.

We hear that Mr. Kyan has abandoned his project of preparing the cordage with the same solution; and he has suggested in lieu of it, a solution of caoutchouc, which, while it protects them against moisture, may serve to make them more elastic.

On the whole the report presented by M. Keraudren to the Academy must be deemed highly favorable to the proposal of Mr. Kyan.

---

### On Spinal Diseases. By Dr. Enz.

It is an admitted physiological position that an injurious impression upon a large nervous trunk is felt not so much at the spot itself where the impression is made, as at the extremities of the ramifications of the nerve. The same remark is strictly applicable to the spinal marrow in its healthy and morbid relations; and it is by keeping this observation steadily in mind, that the scientific physician is often led to suspect and to discover the primary seat of a disease, which may be altogether overlooked and mistaken by the inattentive observer. Numerous are the affections of the thoracic and abdominal viscera, which in their origin depend upon an irritation of some part or other of the spinal marrow; and fortunate therefore it would be, alike to the medical man and to his patient, if in all obscure diseases of these organs the examination of the spinal column was more frequently and attentively made. Of late years, much has been done to elucidate this most interesting department of semeiology, and the readers of this Journal must have derived great and valuable assistance from the copious reviews which we have given of Drs. Griffin, Marshall and Brachet's works in some of our late numbers. There is a long, and in some respects a good paper on this subject in a recent number of one of the most esteemed of the German periodicals (Dr. Rust's Magazine) by Dr. Enz of Wurtemburg, and of this paper we now propose to select its most useful contents.

He informs us that spinal affections (we do not at present allude to distortions or any irregularities of the spinal column) are very frequent in his neighbourhood, the climate of which is damp, chilly and bleak, and where rheumatic complaints are exceedingly prevalent. The forms of disease which irritation of the spinal marrow is most apt, according to Dr. E's experience, to induce, may be arranged thus.

1. Certain diseases which more immediately implicate the nervous system alone; such as mania, melancholia, chorea (one species of which has been designated C. Rachitica) opisthotonos (either alone or complicated with the symptoms of chorea), syncope frequently recurring, or periodic, defects of sight, of speech, and of swallowing.

2. Diseases which affect the nervous and vascular systems simultaneously; such as nervous and intermittent fevers.

3. Diseases which seem to affect chiefly the vascular system; such as some rheumatic ailments, disturbances of the heart and large bloodvessels, and various affections of the other thoracic and of the abdominal viscera. In such cases, the character or type of the prominent symptoms will often vary; but very generally each very marked and decided change of the symptoms will be found to be attended with a change in the particular seat of pain or tenderness of the spinal column.

When the lungs are the parts which are sympathetically affected in spinal maladies, Dr. E. alludes to the great tendency which such sympathetic attacks have to be followed by attacks of decided inflammation of the organs, and ultimately to the deposition of tuberculous matter in their structure.

When the abdominal viscera are the parts which sympathize with spinal irritation, one of the most common forms of disease is that of Colica Damnoniorum, or that species of colic and the attendant systemic disturbance to which those who are exposed to the action of lead are liable. The early symptoms of this disease are often observed to indicate its origin and genuine nature. At first the patient complains of a dull heavy gnawing distress in the bowels, accompanied with flatulence, anxiety and depression. The attacks of pain are usually experienced most severely towards the evening; but as the disease continues, they become more and more prolonged, and remain often during the whole of the day without intermission. After a lapse of some time, one or more of the thoracic or abdominal viscera generally take on an inflammatory action: the stomach and bowels more frequently than the liver, or the lungs. In many cases, the symptoms of the inflammation are very obscure and scarcely to be suspected. Organic disease however may be going on all the time, and the patient, even although his appetite continues good, may gradually be wasting away, and ultimately die in a state of complete marasmus.

In other cases, especially when the constitution is tainted with scrofula, some part of the spinal column often becomes carious and distorted, and death ensues from a consuming hectic fever. The atrophy induced by the operation of lead is often associated with scirrhus of the pylorus, and also with hæmorrhoids.

Another order of diseases which is frequently dependent upon an affection of the spinal cord is that of hæmorrhages.

Thus epistaxis, hæmoptysis, hæmatemesis, and especially menorrhagia may in many cases be traced to this source. 'Often,' writes Dr. E., 'have I been summoned to consultation in obstinate cases of such hæmorrhages where all means of relief failed until once the real cause of the disease was discovered," by a careful examination of the spine.

Dysmenorrhœa, with all its accompanying chlorotic and hysterical symptoms, has very often its origin in some spinal affection; and we have little doubt in our own minds that the reason why the treatment of this disease is so frequently unsatisfactory and inefficient is, that medical men are not in the habit of examining the spine and of directing their remedial measures to the part which is the primary source of all the evil. Dr. E. assures us that, in almost every case of dysmenorrhœa, and in most cases of menorrhagia which have occurred of late years in his practice, he has found that some part or another of the spinal column was more tender under pressure than the rest of it.

So numerous indeed and complicated are the forms of disease which may be induced by spinal irritation, that Hinterberger has justly said, 'that there is scarcely one malady, whose mask it may not wear;' and availing himself of an expressive, but not very correct metaphorical epithet, he has denominated this department of pathology, the cryptogamic. How important therefore it must be to ascertain, in all doubtful cases, whether the spinal cord is at fault or not. Before we close these remarks, we are confident that our readers will be satisfied of the essential advantage which may be derived from such an examination, whenever the nature of any malady is obscure, or its treatment is unsatisfactory. The simple expedient of passing the finger with moderate pressure along the tract of the spine will in most cases be found quite sufficient to detect the latent evil. Whenever pressure is made upon the tender part, the prominent character or symptom of the existing affection will immediately be made more conspicuous. We very generally observe that, in patients who suffer from *constant vertigo*, pressure made over the tender upper vertebræ of the neck induces a boring or penetrating pain felt just behind the glabella of the frontal bone, and this pain is usually attended with a sense of giddiness and confusion; whereas, in cases of *periodic vertigo*, the eighth and ninth dorsal vertebræ are more frequently the sensitive ones, and pressure over them causes a pain in the epigastric region, and a feeling of a fluttering or whirling movement, which commencing there gradually rises towards the head, and then the giddiness ensues. If this examination be made when the patient is standing, the giddiness is sometimes so great that he will drop down in a swoon, if not supported.

A student had been long annoyed with vertigo, weakness of vision, noises in the ears, dyspnœa, cough, palpitations, cardialgia, constipation, sleeplessness, and inactivity of the mental energies, amounting often to confirmed melancholy. On pressing the upper cervical vertebræ, he experienced pain in the forehead; when the pressure was made on the lower cervical and upper dorsal vertebræ, the breathing was much distressed—if lower down, over the sixth and seventh vertebræ, then cough was induced; and, when the space between the 8th and 10th vertebræ were pressed, a gnawing pain in the epigastrium immediately followed.

A boy, 13 years of age, had been for six months suffering, from frequently-recurring attacks of severe colic. These attacks usually came on towards evening, and were often so violent, as to cause him to writhe and roll himself on the ground from the pain. He had voided occasionally some lumbrici, and the symptoms accordingly had been ascribed to vermination. But, as the symptoms did not at all abate after the expulsion of the worms, Dr. Enz suspected that the spine might be at fault. He found, on examination, that sharp pain in the abdomen was induced, whenever firm pressure was made over the lower lumbar vertebræ. Leeches and mercurial ointment were applied over this part, with considerable relief to the symptoms—the tincture of stramonium was used with advantage, both internally and externally, and, ultimately, they were quite dissipated after the application of a blister.

Subsequently, the vision became considerably deranged, and vertigo, and also an inordinate appetite for food, supervened. The cervical and upper part of the dorsal tract of the spine being now found to be rather tender, leeches and a blister were applied there, with the happiest effects. No sooner were these unpleasant symptoms removed, than the young patient began to be affected with dyspnœa and palpitations of the heart, which were sometimes so violent as to force him to start out of bed, and recover the upright position. The middle portion of the dorsal spine being now tender on pressure, a similar treatment, as on former occasions, was adopted, and with equally pleasing results.

This case illustrates the occasionally erratic character of spinal affections—a feature to which Griffin and Hinterbruger have alluded in their memoirs.

A woman consulted Dr. E. for a troublesome cough and dyspnœa, which had existed for upwards of a twelvemonth. These symptoms were accompanied with a sense of weight in the epigastrium, vertigo, sleeplessness, and tendency to extreme depression of spirits. On examining the spine, that portion of it between the fifth and the tenth dorsal vertebræ was found to be tender on pressure. It was leeched, and the mercurial ointment was afterwards applied to it night and morning. Subsequently, three successive moxæ were established over the most tender points, and pills, consisting of calomel, jalap, extract of nux vomica, and of the p. gratiolæ, were ordered to be taken for some time. These means greatly mitigated all her distressing symptoms, although a complete cure was not obtained.

But, in many cases the symptoms are much less distinct than in those now related, where the tenderness of a particular part of the spine very obviously corresponded with a particular train of symptoms. Sometimes a considerable extent of the spine is affected at the same time, and then not only one organ, or one set of organs sympathises, but several organs, apart from and independent of each other, are involved in the diseased action, and the character of the case is, therefore, very complicated and obscure. The following may be taken as an example of such a state of things.

A man, 57 years of age, was seized, in Feb. 1832, with an inflammation of the neck, which caused great stiffness and pain in the part. Without any warning he expectorated, or rather vomited, a large quantity of coagulated blood, and Dr. E. was summoned to his assistance. He found him suffering from a violent spasmodic cough, great dyspnœa, dreadful pain at the scrobiculus cordis, a sense of burning in both hypochondria, and a cutting pain in the hypogastrium. The bowels had been constipated for the preceding four days; a degree of strangury also existed, and the lower limbs and scrotum were enlarged, from œdematous effusion. The patient complained much of general coldness, and he felt, to use his own expression, as if cold water was poured down his back. These symptoms induced Dr. E. to examine the spine. The extent from the 3d to the 6th cervical vertebræ was very painful on pressure; from the 6th cervical to the 3d dorsal, it was free from tenderness; but lower down, as far as the 7th dorsal vertebra, it was again painful when pressed upon. The rest of the spine, with the exception of the three lowest lumbar vertebræ, was not at all tender. Leeches were applied, and then some mercurial ointment was rubbed in over the parts which were tender. Several doses of active purgatives were administered. The symptoms were decidedly abated by these means, and the sense of general coldness was much diminished. But every now and then the cough and dyspnœa, or the severe abdominal pains, returned, continuing generally for several hours at a time with great severity. Blisters were used with only partial relief. An emulsion, containing oil of turpentine (so useful in acute neuralgia) and sulphate of soda, speedily mitigated the distress in the breathing so much, that in the course of three or four days he was able to resume his customary employment. After a lapse of some months, he experienced a relapse of the cough, and of the œdematous swellings; and these symptoms were now accompanied with great rheumatic pain of the scalp, and with urticaria over the whole surface of the body. Complete relief was again obtained, by the administration of the spirit of turpentine.

In the history of spinal irritation, it deserves to be noticed that, in some rare cases, there is not a strict or uniform correspondence between the suffering organ and the affected portion of the spine; or, in other words, that the disease sometimes exhibits itself, not in the organ or organs which derive their nerves from, and which usually sympathise with, that portion of the spine which is tender on pressure—but in other organs, at a distance from and unconnected with it. Thus, Dr. E. has observed a severe and most obstinate colic, when the lower cervical and upper dorsal vertebræ only were tender on pressure, and this colic was cured by the inunction of the mercurial ointment, and by the application of blisters over the tender region: and, again, in more than one instance, he has seen a long-protracted periodic vertigo coexistent with, and seemingly dependent upon, an affection of that portion of the spine between the sixth and eighth dorsal vertebræ, a cure being effected by the repeated blistering of the part.

This want of uniformity between the set of existing symptoms and the portion of the spine affected, is most conspicuous in mental diseases. Sometimes the entire extent of all the cervical and dorsal vertebræ is exceedingly tender on pressure; at other times, only one part of this extent (and perhaps the most frequently affected is the interscapular portion) indicates this sensitiveness. But why, in the latter case, a cough, and, in the former, a complication of morbid phenomena is not always present, is as difficult to be explained, as that an affection of the cervical vertebræ should give rise, in one instance, to a constant vertigo—in another, to some defect of vision, and in a third, to dysphagia. These are among the mysteries of spinal pathology.

Hitherto we have alluded to the symptom only—that of tenderness of some of the vertebræ on pressure—of spinal affections. But there are other signs or symptoms, which, although less obvious and characteristic, are very generally present whenever any portion of the spinal cord is at all disturbed by disease. There is a remarkable weariness and lassitude of the whole body; the patient is fatigued on the slightest exertion, and he feels, when he exerts himself, as if his knees gave way underneath him. At some part of the abdomen, there is often a constrictive pain, and this usually distresses the breathing, and gives rise to frequent yawning or sighing, or even sneezing. In most cases, unless indeed the affection is confined to the lowest part of the spine, there is a decided tendency to attacks of shivering, syncope, and of palpitations of the heart. The appetite is very often not at all impaired, at least for a length of time; sometimes it is unusually active. Any irritating food, however, and more especially spirituous drinks, add to the general uneasiness of the patient, although he may remain all the time free from any well-marked febrile irritation, and be able to attend to his accustomed employments. As a general remark, it may be stated that the vascular system does not usually indicate any decided disturbance of its actions for four, or even six or ten months after the commencement of the spinal malady, unless indeed the general powers of the system began speedily to decay from atrophy and marasmus. The varying character and intensity of the symptoms which we have enumerated above, give rise to numerous modifications of spinal disease. The pains felt in the abdomen may be either acute or dull; sometimes they are burning, or tearing, or boring—at other times they are felt as if the short ribs, or the parietes of the abdomen, were dragged away from their attachments. When the epigastrium is the seat of the distress, the patient will often be found to complain of an unutterably distressing sensation, as if a heavy body there was suspended upon a thin thread, and every moment threatened to sink down.

The cough is very generally of a convulsive character, recurring in paroxysms of great severity; and the distress of breathing is sometimes so great, that the patient is in danger of immediate suffocation. Numerous are the cases of asthma which are primarily owing to irritation of some portion of the spinal marrow.

In female patients, the catamenia are almost always very irregular in the course of spinal diseases; usually they are scanty—but an occasional attack of menorrhagia is not unfrequent, especially when there has been a complete cessation of the discharge during several months previously. The action of the bowels is usually more or less torpid; this state of torpor being, however, occasionally interrupted by a diarrhœa, of some days' standing. The excretion of the urine is not less frequently affected. A degree of dysuria or ischuria is often present, and sometimes there is an incontinence, giving rise to a constant dribbling of the water. Some authors have asserted that there is generally a deficiency in the quantity of the urine secreted; but this observation is not confirmed by Dr. E.'s experience.

In addition to these more prominent symptoms of spinal irritation, there are others, of minor importance indeed, but which, when present, render the diagnosis more clear and satisfactory. To this class belong the flying pains and twitchings of the extremities, more especially in the calves of the legs, and in the fingers of one or of both hands. Œdema of the feet is a symptom of occasional occurrence. The sleep is very generally short, uneasy, and interrupted. In some cases, there is an almost total deprivation of sleep for several weeks at a time.

The perceptions of the different external senses are apt to be more or less impaired or perverted. Thus the sight, the hearing, the taste, the smell, and the touch, are all occasionally subjected to various irregularities or depravations. Here we may allude to a not uncommon symptom of spinal irritation, that species of headache to which the appropriate appellation of "clavus" has been given. The headache is sometimes, however, much more diffused than in the ordinary "clavus," for the whole extent of the scalp is frequently affected with a most distracting pain. In the neck, along the tracks of the nervi vagi, the patients often complain of a stretching or dragging pain, as if the windpipe was forcibly blown out. The abdominal muscles are not unfrequently affected with partial spasms, so as to cause the surface of the belly to be drawn into numerous irregular hollows and elevations. We have already alluded to the sensation which is often experienced, as if cold water or ice were laid on some part of the spine. The "decubitus" of the patient deserves to be noticed. He usually lies on his back, and the effort to turn his body round causes uneasiness, or even positive pain, at some part or another.

The skin, provided no fever is present, is commonly cool, and of an unhealthily pale or yellow colour. In some cases, especially when the disease is acute, and in which the irregular fits of shivering already noticed are apt to occur, the unexpected phenomenon of the skin being warm and moist during these cold fits, and, as the shivering abates, of its becoming again marble-cold, is not unfrequently met with. Whenever the vascular system is once fairly involved in the morbid process, a marked change in the symptoms is observed. The patient is no longer able to attend to his employment, or even to move about without great distress: often indeed he is obliged, from this period, to confine himself to bed. His appetite decays, and all his energies gradually waste away. He begins now to feel sharp, burning pains in some part of the spine, greatly aggravated on any exertion. If the chest has been the part chiefly affected, the cough becomes more troublesome, the expectoration increases in quantity, and, at length, a confirmed consumption may be induced. In other cases, the encephalon is the part which suffers most severely, and then a state of raving delirium, or of slow feverless insanity, comes on. Dr. E. has related several cases of mental disquietude, varying in degree from temporary melancholy to confirmed derangement, which, he thinks, was owing mainly or altogether to irritation of the spinal cord. The narratives of these cases certainly do not, in our opinion, warrant such a conclusion; and we shall, therefore, abstain from detailing their particulars.

The following case of chorea rachitica is more worthy of notice, and more especially as it will afford to our readers a fair specimen of the therapeutic practice of an enlightened German physician.

A girl, 13 years of age, was brought in Sept. 1832, to Dr. E. in consequence of convulsions, with which she had been afflicted for the preceding fortnight. He could learn no further particulars of the case. A mixture, containing four grains of calomel and of the flowers of zinc, a drachm of gum arabic and four ounces of black cherry water was ordered; of this, a tablespoonful was to be given every two hours, and the spine was ordered to be rubbed night and morning with a liniment, composed of camphorated hartshorn oil, spirit of turpentine, and extract of henbane. For the following sixth months, little amendment took place. Powders, consisting of calomel, jalap, and extract of valerian were prescribed. The frequent strangling cough, the half-bent state of the fingers and hands, the tremulous movements of the head, the tenderness of the epigastrium on pressure, and the throbbing pulsations of the abdominal aorta—all these symptoms confirmed Dr. E.'s suspicions, that the cause of the evil was in some part of the spinal marrow. When the little patient attempted to speak, the corners of the mouth were distorted, and often indeed the jaws were spasmodically closed, and an attack of partial opisthotonos ensued. Her gait in walking was trembling and unsteady, so that, unless she was supported, she would be every now and then falling down. Deglutition also was at times difficult, and even impracticable. On examining the back, almost the whole extent of the spine, more especially the cervical and interscapular portion, was found to be very tender on pressure. The calomel, jalap, and valerian powders were ordered to be continued, and a liniment, composed of mercurial ointment and of extract of opium, was rubbed along the spine every night and morning; premising, however, the application of some leeches over the most tender points.

By steadily persevering in this treatment for the space of six weeks, a decided improvement was obtained; and ultimately the girl was quite restored to health.

A case also of opisthotonos, induced by exposure to cold and damp, from lying all night on the wet grass, is related. The muscles of the jaw were partially affected. The attacks of spasm occurred usually towards evening and at night, and were accompanied with violent pain in the right hypochondrium, and with great dyspnœa. As soon as the patient, a middle-aged woman, felt the paroxysms coming on, she was obliged, if reclining, to spring up, and to hold by two or three people, to maintain herself in the upright position. On examining the spine, some of the middle dorsal vertebræ were very tender on pressure. Leeches and the cupping-glasses were applied repeatedly over this part, and the mercurial ointment was rubbed in afterwards. These, along with some internal remedies, were used steadily for several weeks, and ultimately with an entire relief of every symptom.

Several cases of obstinate vertigo, constant as well as periodic, are related, in which a tender state of some portion of the spine seemed to be the real cause of the mischief, seeing that cures were effected by an appropriate treatment, directed to the removal of the spinal affection. Dr. E. has generally observed, that the constant or abiding form of vertigo is accompanied with a tender state of the upper cervical vertebræ—and the periodic form, with a tender state of the lower cervical, or of the upper dorsal vertebræ. The following case of vertigo, associated with amblyopia and dysphagia, is interesting.

A countryman, having been much exposed to wet and cold, began to experience sharp flying pains in both lower extremities, and also in the hypochondriac and epigastric regions. The pain felt in the epigastrium was accompanied with a dull oppressive pulsation, and with excessive languor and oppression. The bowels were much confined, and the patient suffered from occasional vertigo. This last-named symptom was usually most severe towards the evening, and during its presence the sight was so imperfect, that he could scarcely distinguish any object. For upwards of half a year, the health of this man became gradually more and more impaired. He began to experience great difficulty in swallowing; his hands were occasionally affected with convulsive twitchings, and a most distressing strangury was added to his miseries. The bowels were most obstinately costive; at times he saw double; at other times he was nearly blind. The spine was examined by Dr. E., who found that all the cervical vertebræ were very tender when pressed on. By the use of local depletions, of frictions with mercurial ointment, and of blisters, associated with the internal administration of calomel, jalap, and valerian, this man was eventually restored to perfect health.

Dr. E. has been able to trace in several cases the dependence of amaurosis on a spinal affection. The following may be taken as an instance of this sympathy.

A man began in his twenty-fifth year to experience great weakness of vision. In the course of a few years he became completely amaurotic: the strongest light made no impression on the eye. Besides the blindness, he suffered from a constrictive pain in the region of the diaphragm, and from great weakness and occasional tremors of both the upper and the lower extremities. All the visceral functions, however, appeared to be healthily performed. The second, third, and fourth cervical vertebræ were found to be tender on pressure. The middle dorsal, and one or two also of the lumbar vertebræ were tender.—Leeches first and then camphorated volatile mercurial ointment were applied to the affected portions of the spine; and occasional doses of calomel and jalap were administered. A blister also was applied to the nape of the neck, and an eighth of a grain of strychnine was sprinkled daily on the raw surface. The sight having returned partially under this treatment, the dose of the strychnine was raised to a fourth of a grain. Within a month after the commencement of Dr. E.'s attendance, the patient could distinguish the light of a candle, (which, we are informed by our learned author, is in that part of Germany a good beechen stick, full four feet long!) After the blister had healed up, several moxæ were established on the nape of the neck. Blisters were subsequently applied on the temples, and their surfaces sprinkled with minute quantities of strychnine. Eventually the sight was restored so much that he could distinguish, indistinctly indeed, the outlines of most objects.

The following case of mental disease, presumed to be dependent on spinal irritation, is selected from a multitude of less interesting examples.

### Partial Insanity, without Fever.

A man, of a firm robust constitution, began to exhibit, in the Autumn of 1830, signs of insanity, and oppression of all his physical and mental energies. He suffered occasionally from severe colic, especially after drinking wine, or other liquors. In the March of the following year, he was seized, after exposure to wet and cold, with an inflammatory fever, which was accompanied with delirium. As the febrile symptoms abated, the insanity returned in an aggravated degree. For the six successive weeks he did not sleep a single minute. Dr. E. was led to suspect that the spinal cord was somehow at fault. Firm pressure over the lumbar and lower dorsal vertebræ excited no uneasiness; but pressure over the upper dorsal vertebræ immediately caused him to wince and cry out. The cervical vertebræ might be pressed upon without causing any inconvenience. The tender region was freely leeched, and afterwards anointed twice a day with mercurial ointment. Calomel, united with minute doses of cocculus indicus, was administered inwardly.

The restlessness was in some degree subdued, after this treatment had been pursued for a short time.

A blister was then applied, and small doses of emetic tartar, to be frequently given, were substituted for the calomel: purgatives also were occasionally used.

By following out this plan of medication, this man was eventually restored to at least a much better state than he had been in for some years, and even enabled to resume his occupation as a common labourer.

Several other similar cases are adduced by our author.

In all of these, tenderness of some part of the spine was a prominent symptom, and relief was invariably derived from local abstraction of blood. But as most of these cases were of recent standing, and the other therapeutic means were powerful and steadily continued, it is impossible to determine, how much of the ultimate benefit is to be attributed to the leechings. It may be worth while to mention a formula, which, he tells us, is often of the greatest use in cases of chronic illness connected with disease of the spinal marrow.

℞. Oxymur. hydrarg. gr. ij.

Extract. nucis vomicæ, gr. viij.

——— Aconiti, gr. viij.

——— Glycirrh. ℈j. M. in pil. xx. divid. ii. q. n. et m. sum.

These pills Dr. E. recommends strongly in neuralgia, chronic rheumatism, &c.—*Rust's Magazin für die gesammte heilkunde.*

There are some valuable hints and suggestions in the preceding observations of Dr. Enz. Like most authors on any favorite topic, he carries his speculations too far, and ascribes an inordinate share to spinal irritation in the production of diseases; for example, in referring most cases of dysmenorrhœa to this source. Some cases have very recently come under our own notice, which very aptly illustrate the importance of attending to the spine in some obstinate and obscure cases. One of these is more than ordinarily interesting. A youth had for nearly sixteen years been subject to frequent returns of dreadful palpitations of the heart. These attacks, though appalling at the time, usually passed away in from a quarter of an hour to an hour, or rather more, leaving the patient only somewhat exhausted. His general health, although delicate, did not suffer much; he was able to take very active exercise, and could ride upwards of forty miles a day; and it was generally observed, that the more exercise he took, the less subject was he to the palpitations. The inducing cause of these attacks was obscure; for often would they come on when he was quite quiet, and even when asleep. The greatest relief was obtained by pressing the chest very firmly against any firm object, as a stool, the post of the bed, &c. while an attendant kept beating the back with his fist.

The case had been examined by many of the most able physicians in London and Edinburgh, and had been regarded by all of them as one of organic disease of the heart. That the heart was greatly hypertrophied they all agreed, but whether the valves were diseased, was disputed by some. (We never had an opportunity of examining the case ourselves, nor did we ever hear what were the stethoscopic signs.) The patient himself had discovered that pressure upon the lower cervical and the upper dorsal vertebræ always induced a sharp pain in the region of the heart, and a tendency to an attack of the palpitations, and he used to maintain against the opinion of all his doctors that the heart itself was not diseased. An attack of bronchitis last December proved fatal. On dissection, it was found that the heart was quite normal, with the exception of a very inconsiderable hypertrophy of its walls. There was a slight lateral curvature, with the convexity to the left side, of the lower cervical and upper dorsal vertebræ.—*Rev.*

---

### On Secondary Abscesses, by Dr. Nasse of Bonn.

Under the term "secondary abscesses" are included all such purulent deposits as are formed in a part or parts (generally visceral) remote from, and unconnected with, the seat of a primary abscess. Secondary abscesses are found to occur most frequently after wounds of the head, amputations, fractures of bones, operations on the womb, or on the mamma, the removal of hæmorrhoidal excrescences, and all wounds or injuries of veins. The internal organs, in which such abscesses are by far the most frequently found, are the lungs and liver. It is rare to meet with cases in which secondary abscesses exist in any internal part, without the lungs being at the same time similarly affected. In the bags of the pleura and peritoneum, secondary purulent effusions sometimes take place:—the spleen too is occasionally the seat of secondary abscesses. The pleura indeed very generally becomes sympathetically inflamed, whenever any secondary abscesses are formed in the pulmonary texture; and when so affected, it soon begins to exude a puriform fluid. The various synovial sacs are very frequently the seats of purulent deposits. Some portions of the cellular tissue, and especially that in the mediastina, are occasionally, but much more rarely so. The substance of the brain also is sometimes, although very rarely, the nidus of secondary abscesses. Even the thyroid gland and the cornea of the eye have, in a few instances, been the suffering organs.

Hitherto the German writers on surgery have strangely overlooked this curious and important subject of pathological research, although their attention might have been drawn to it by the remark of some of the French authors of last century in reference to the secondary effects of injuries of the head.

Among the English surgeons, the late Mr. Rose of St. George's Hospital, is among the first who deserves well of his professional brethren, by having illustrated this subject in a paper published in the 14th volume of the Medico-Chirurgical Transactions of London. Subsequently M. Dance, in the t. xix. of the Archives Génerales, and Cruveilhier, in his Anatomie Pathologique, have added many interesting details to our stores of knowledge. They have pointed out the very frequent connexion of secondary abscesses with inflammation of the veins.

Dr. Nasse informs us that he has had for several years past many favorable opportunities of witnessing cases, wherein secondary abscesses were discovered on dissection. In tracing the pathogeny of these lesions, there are, says he, three stages to be observed. The first may be characterised as that of blood-infiltration into the affected tissue;—the second, which is in fact the commencement of the suppurative change, is distinguished by the affected part acquiring a yellowish colour, and a soft easily lacerable consistence, so that it breaks down between the fingers, while the surrounding textures become more vascular and indurated than in health; and the third or last stage is that of the infiltration or of the circumscribed deposition of purulent matter. A peculiar feature of secondary abscesses is the well marked limitation (scharfe begränzung) of the sanguineous, as well as of the purulent infiltration in the affected tissue. The pus deposited is usually thick, yellow-coloured, and is not always quite uniform in quality. Sometimes it is firm and almost solid, and then it is not unlike to soft tuberculous matter. In the centre of such pseudo-tubercles, some fluid pus is usually found.

So much for the general features of secondary abscesses. We shall now briefly examine their particular characters in reference to individual organs.

In the Lungs, the organs most frequently affected, the earliest appearances of secondary purulent deposits are dark-red circumscribed spots, or patches, not unlike to what we observe in those parts of the pulmonary tissue which have been at a former period the seat of apoplexia pulmonum, induratio hæmoptyca of Laennec. In the course of a short time, the red colour of the spots becomes streaked or marbled with numerous yellow lines or points, such as we observe in the grey hepatization of a portion of lung, which has suffered from inflammation. As the structure of the part becomes more decidedly and uniformly yellow, it loses its firmness, and is readily lacerable, breaking down on pressure, and presenting a remarkable degree of porosity, after it has been squeezed between the fingers. When washed, it has a grey colour, and is then like to, but much softer than, a portion of grey-hepatised lung. If thrown into water, it usually sinks at once to the bottom, or hangs suspended some way below the surface. The effects of maceration satisfactorily demonstrate that the purulent matter is actually infiltrated into the pulmonary texture. The pulmonary tissue in the immediate neighbourhood of, and forming the walls of secondary abscesses is very generally thickened, and appears to be more highly vascular than in a state of health. The infiltrated matter, as it increases in quantity, and in consequence also of the breaking down and solution of the interposed tissue, gradually becomes more and more completely collected into one uniform abscess, until at length there is formed a perfect sac of matter, the walls of which sac are firm and resisting. This matter, when examined on dissection, is however usually mixed with ligamentous or cellular shreds (häutigen theilen) floating in it. The number of secondary abscesses in an organ differs greatly in different cases. Sometimes we meet with one large abscess only; at other times there are not fewer than twenty. Their size too is quite as various; some being not larger than a kidney-bean, while others equal a hen's egg. Dr. Nasse has occasionally seen a lung exhibiting in different parts of its structure, secondary abscesses in all their various stages, from that of simple lobular engorgement and discoloration, to the formation of a perfectly encysted deposit of pus. Secondary abscesses are found most frequently in the lower lobes of the lungs, and in the lower portions of these lobes. Once only has our author met with a considerable number of them in the upper lobes. They are more common in the posterior than in the anterior portions of the lungs. According to the researches of Mr. Rose, the greater number of pulmonary secondary abscesses are superficial, immediately under, or at least very near to, the investing pleura, which is then usually uneven, discoloured, streaked with numerous minute vessels, and often covered with an exudation of coagulable lymph. Sometimes however they are found in the very central parts of the lobes. The pulmonary tissue surrounding the walls, and indeed the walls themselves of secondary abscesses, are sometimes quite gangrenous, and then the contained matter is always offensive and fætid. Cruveilhier has described minutely the appearances which he has found in the neighbouring bloodvessels. They are usually plugged up with coagula. In these coagula he has frequently met with minute depositions of pus.

When secondary abscesses are formed in the Liver, their development is indicated at first by dark brownish-red blood-patches (blut-flecken). In these, as the purulent matter is gradually deposited, the minute grains or acini of the hepatic substance become of a yellowish colour, the affected portion presenting then the appearance of some sorts of granite. As the pus increases in quantity, this marbled appearance becomes more indistinct, and the part becomes of a uniformly yellow colour, until at length the solid matters are quite broken down and dissolved or absorbed, and an abscess more or less completely encysted is formed. Dr. Nasse has found in a liver as many as forty secondary abscesses, varying in size from that of a pea, to that of a fowl's egg. Cruveilhier mentions a case, in which there was only one large abscess, occupying a considerable extent of one lobe. Bertrandi was of opinion that these abscesses are more frequent on the convex, than on the concave side of the liver. They are met with in all parts of the viscus; immediately under its tunica propria, as well as deeply imbedded in its substance. The contents of secondary abscesses in the liver are described as being usually " yellow coloured, coarse-grained, flocculent, adhering to the parietes of the abscess, and not unlike to the parenchyma of the liver, after it has been macerated for a considerable time." Laudable pus is very rarely or never found in such abscesses. The veins contiguous to the abscesses often contain pus mixed with coagula of blood.

The changes which precede the formation of secondary abscesses in the Spleen have not been satisfactorily investigated. We have already stated that abscesses are of rare occurrence in this viscus. When purulent matter has, in such cases as we are now alluding to, been discovered in the Joints, the lining synovial membrane very often, nay generally, exhibits no traces of inflammation: it would seem as if the purulent effusion had been poured out as a simple exhalation, without any previous inflammatory action.

Dr. N. alludes to the discrepancy of opinion between the French and English surgeons, respecting the relative frequency of secondary hepatic abscesses after injuries of the head:—the former representing it as a very common accident, while the latter in their writings do not frequently allude to it. His own observations agree with those of the late Mr. Pott, and of Sir B. Brodie, that there is in fact no greater sympathy of morbid action between the head and the liver, than between the head and some other parts, such as the lungs or peritoneum. Valsalva informs us that in all his examinations of patients who had died after injuries of the head, the liver was only once, and the lungs were four times found to be affected; and Klein is of opinion that the liver is more apt to be the seat of secondary abscesses after lesions of other organs, than of the head.

The researches of most recent pathologists confirm these views in opposition to the long-taught doctrines of the French school, and prove that the lungs are more frequently affected with secondary suppuration than any other parts of the body, and that whenever any other part is so affected, the lungs almost always suffer at the same time.

Mr. Rose however has reported one case, in which the liver only was found to be the seat of secondary abscesses; all other organs being sound. Such a case is of very rare occurrence; and we may therefore re-affirm with perfect confidence that, whenever a secondary abscess is discovered in the liver, some other organ, and most frequently the lungs, will be found to be similarly affected.

Wounds, amputations, fractures, &c. of the lower extremities are observed to induce secondary abscesses in the lungs much more frequently than either in the liver alone, or in the lungs and liver together—in the proportion of at least 11 to 3. As examples of hepatic secondary abscesses existing alone and unassociated with any similar lesion in other organs, we may refer to a few cases published by the English surgeons Hennen, Lawrence and Rose, and also by Cruveilhier in his Anat. Path. 'Maladies du foie.' According to some experiments on brutes recorded by the last-named author, mechanical irritation of the vena cava inferior is apt to be followed by the formation of secondary abscesses, and such abscesses, he says, are invariably found in the pulmonary structure. After injuries of the lower extremities, the hip and knee joints are not unfrequently the seat of purulent effusions. Occasionally the joints more remote from the seat of the injury are similarly affected. Our author has discovered in one or two instances secondary abscesses developed in muscular tissue:—the diaphragm is perhaps more liable to this lesion, than any other muscle. The pericardium, peritoneum, intestinal mucous membrane, and even the brain are not exempt from a liability to secondary inflammation, and suppuration, after injuries of the extremities.

Dr. Nasse informs us that, after wounds and other injuries of the upper extremities, the liver is more frequently affected than the lungs—an observation which we should certainly not have 'à priori' expected. Abscesses have been repeatedly found after such injuries in the former viscus, while the lungs were altogether free from them. The pleura may become inflamed and may exude a purulent effusion, without the pulmonary parenchyma being decidedly involved in the morbid process.

Mohrenheim has occasionally discovered an effusion of purulent matter into the abdominal cavity; and Lawrence, once found matter in the kneejoint, without any other co-existing lesion, after a fracture of the os humeri. The shoulder-joint itself is very frequently the seat of such effusion. The pericardium, and even the substance of the heart itself have been found to exhibit traces of secondary inflammation and suppuration after injuries of the upper extremities.

Mr. Travers mentions two cases in which he found purulent matter in the jugular veins of horses—in one, the inflammation had extended upwards to the brain, and in the other, it had descended to the lungs and pleura. Gaspard performed many experiments on dogs, to ascertain the effects of injecting into their bloodvessels purulent matter taken from the human subject:—the lungs, heart and intestinal canal, in numerous instances were found to exhibit signs of inflammation.

It is proper to state that Velpeau, Majendie, Prevost and others, have repeated these experiments, and that the results obtained by them have been very inconstant and indecisive.

We have not yet alluded to the effects, which disease in organs whose veins belong to or communicate with the Portal system, has in inducing secondary abscesses in distant parts. Suppuration occurring in the uterus, the bladder, or the rectum, is followed sometimes by the formation of secondary abscesses in the liver only; in other cases the lungs alone, and in a third set of cases, the lungs and the liver simultaneously have been affected. More rarely the spleen, the brain, and the joints are in such cases the parts sympathetically diseased.

The *symptoms* which usually accompany the development of secondary inflammation and suppuration, although obscure, and necessarily varying according to the organ chiefly affected, indicate in all cases a great and decided constitutional disturbance.

It is commonly at some period between the tenth and the twentieth day after the receipt of the injury, or the formation of the internal disease, that the secondary symptoms are first observed. In some cases, however, the date of their appearance is later—not until the fourth or fifth week. Indeed, as long as any primary suppuration exists, a sympathetic morbid action may be developed at some remote part, this action giving rise ultimately to the formation of secondary abscesses.

No age or constitution is exempt from this very serious complication; but the dissipated and unhealthy are most exposed to its attacks. The earliest indication of the mischief is usually the occurrence of hectic chills, followed by high vascular excitement, and by more or less profuse perspirations.

Indeed, it may be asserted with much truth, that whenever these unpleasant symptoms come on at any period between the second and fourth or fifth week after the receipt of an accident, or the infliction of an operation, the surgeon has too much reason to suspect an incipient suppurative process in some part or another. The frequency of the venous trunks and branches in the neighbourhood of the primary lesion being found on dissection inflamed, and containing pus, might lead us to expect that the phlebitis should be often discoverable during life, at least in those parts where the veins are superficial. But this is rarely or never the case. The wound, if external, may have taken on an unhealthy aspect, the lips having become inflamed, and the discharge scanty and ichorous, and the affected limb may have assumed an œdematous enlargement (a tolerably certain sign, in such cases, of an obstructed venous circulation); but the veins themselves nevertheless are not, at least generally, to be felt like hard and unyielding cords, as in ordinary cases of phlebitis. When the lungs are the seat of the sympathetic suppuration, there are, in addition to the pyrexial symptoms already noticed, cough, constriction of the chest, dyspnœa, and great anxiety. Neither percussion nor auscultation will assist our diagnosis much, the inflammatory and suppurative changes being too circumscribed, and too scattered, to affect materially the normal sounds, unless indeed effusion into the cavity of the pleura has already taken place. In some cases, the pulmonic symptoms are exceedingly obscure, even when large and numerous vomicæ are found throughout the lungs after death.

Dr. N. alludes to such a case, in which the patient had not exhibited a single thoracic symptom. On dissection, several abscesses were discovered in the lungs, and also in the substance of the brain. Equally uncertain may be the symptoms, when the disease is seated in the liver; sometimes there is a fixed pain in the right hypochondrium, and this pain may be accompanied with an icteric discolouration of the skin, and with an unhealthy state of the alvine evacuations; at other times, none of these characteristic signs are present, and yet the viscus may be found as seriously injured as in the former case. Whatever may be the condition and progress of the local distress, the constitutional disturbance invariably increases in severity, and becomes more and more alarming. The patient falls into an adynamic or typhoid state—the tongue becomes parched and brown—the skin hot or wet with perspiration—the pulse rapid, feeeble, and irregular—the patient is restless and soon exhausted, and delirium and stupor usually precede, for a day or two, the fatal event.

With respect to the presumed etiology of secondary abscesses, it may be interesting to revert to the doctrines entertained by some of the older French surgeons, on the cause of that sympathy of the liver with the head, which disposed the former organ to be so frequently affected after wounds and other injuries of the brain, or of its hard and soft envelopes. Bertrandi proposed the following explanation. When any part of the head is inflamed, the blood is sent through the carotids and their branches, and necessarily returns also by the jugular veins in larger quantity, and with greater force, than usual. The descending current in the vena cava superior, meeting with the ascending current in the cava inferior, obstructs it to a certain degree, and thus causes a degree of pressure backward upon the blood in all the veins which enter into the inferior cava; the hepatic veins being large, and situated near to its termination, feel this pressure the most, and, in consequence of the congested state of the liver thus induced, inflammation of its substance is excited. A somewhat similar notion was entertained by Ponteau, who referred all sympathetic inflammations to some local obstruction and congestion of blood.

Chopart, Dessault, and, more recently, Richerand, have rejected the idea of any secondary or sympathetic origin of the abscesses we are now speaking of, and maintain that they are always the result of some positive injury of the organ, received at the same time with, or subsequently to, the conspicuous and apparently more serious accident, which has been deemed the primary one. Thus, with respect to hepatic abscesses occurring after injuries of the head, they refer them to the contusion or concussion of the liver at the time of the accident. Well may Dr. Nasse express his surprise at such sentiments, from men so practical as the great Dessault, of last century, and the able and acute Richerand, of the present. May not injuries of the head be received without any concussion of the rest of the body? and are such cases invariably exempt from the occurrence of secondary abscesses? And how, according to this doctrine, shall we explain the formation of pulmonary abscesses, so frequently found in the bodies of those who have died after amputations, and other operations? But it is surely quite unnecessary to urge more objections.

A more recent hypothesis, as to the origin of secondary abscesses, is founded on the assumption that, in all cases, tuberculous deposits have primarily existed in the sites of these abscesses, and that the deposits have been converted into purulent collections, in consequence of inflammation induced by a febrile excitement of the system. The entire absence of unchanged tubercles in other portions of the diseased organs—the occasional extreme rapidity of the formation of secondary abscesses, and the characters of their contents, are powerful objections to the truth of this theory.

Bichat, Boyer, Bricheteau, and others, are disposed to attribute secondary abscesses to a purely nervous sympathy between the seat of the primary lesion, and the organ secondarily diseased. In reference to the effects of injuries of the head on the liver, the nervous sympathy existing between these parts is, they say, indicated by the bilious evacuations and the icteric discoloration of the surface, which so frequently supervene on wounds and contusions of the head, and also by the results of experiments, in which the nervi vagi have been tied or otherwise injured. Unfortunately, however, the very appeal to these experiments is somewhat unfavourable to the cause they are adduced to prove; for the lungs and pleura are, much more frequently than the liver, found to be inflamed after the tying of the nervi vagi.

Some of the best pathologists of the present day, finding that all the preceding doctrines are quite untenable, have adopted the opinion, that secondary abscesses are in all cases owing to the direct absorption of purulent matter from the seat of the primary lesion into the sanguineous circulation, and to its subsequent deposition in organs at a distance from it. An argument strongly urged in favour of this opinion is, that the veins in the neighbourhood of abscesses, and of suppurating wounds, are often found to contain genuine pus. Velpeau, Cruveilhier, &c. have found pus in the inferior cava, and in the right cavities of the heart, in patients who have died after amputation of the leg.*

Purulent matter has been found in the lymphatic vessels by many anatomists; but it does not seem probable that it enters the circulation in this manner, not only because genuine pus has never been discovered in the thoracic duct by such pathologists as Andral, Dumas, and Gaspard, (and only once or twice by Velpeau,) but also because it would necessarily be arrested in its progress at the conglobate glands.

Notwithstanding these objections, the phenomena of some cases, where collections of matter have very suddenly disappeared, and the urine has thereupon been charged with a quantity of semi-puriform deposit, are explicable only by supposing, that the pus has been taken up by the absorbents. Chelius and Treviranus relate the particulars of an interesting example of empyema, in which the urinary deposit was observed to be remarkably similar to the fluid which was discharged from the thorax. In such cases, it is probable that the thinner parts only of the pus are directly absorbed, as the globules of ordinary pus are said to be too large to pass freely through the ordinary lymphatics, and, as some abscesses which have become very suddenly dispersed, have been found to contain a substance not unlike to half-softened tuberculous matter. When quicksilver has been injected into the veins, and the animals have died some time after the experiment, small abscesses, presenting much of the appearance of tubercles, have been found around the metallic globules discovered in the lungs and in the liver.

On the whole, it appears to us the most rational and best-supported etiological doctrine of secondary abscesses, to trace their connexion with, if not their immediate dependence upon, a diseased condition of the veins immediately adjacent to the seat of the primary lesion.

After fatal amputations, and injuries of the head which have been unfortunately followed by the formation of secondary abscesses, the veins leading directly from the wound, in the former case, and, in the latter, the veins and sinuses of the brain and of its membranes, have been almost always found inflamed, and containing pus. The re-

---

* It deserves notice, that coagula of blood, containing pus, have been found in the right cavities of the heart by Laennec, Legroux, Hache, and others, although there was not a "foyer" of suppuration in any part of the body. In all these cases, the blood itself was in a diseased, degenerated condition. Flandrin (*Rêvue Med.* 1831) found pus in a coagulum in the left ventricle, in a woman who had died, after a long agony, from hypertrophy of the heart and inflammation of the lungs.

sults of very numerous dissections have satisfied Dr. Nasse, that phlebitis in some part of the body, and most generally in the immediate vicinity of the original disease, is, in the majority of cases, an accompaniment of secondary abscesses. Often, when this morbid appearance has not been discovered, Dr. N. is of opinion that it might have been so, had the bloodvessels of the bones been minutely examined; and he attribntes to this omission the circumstance of his meeting with fewer cases now than formerly, where no traces of phlebitis have been discovered in examining the bodies of those who have died with secondary abscesses. To prove the importance of investigating the veins of the bones, it is worthy of notice that Cruveilhier, after injecting some quicksilver into the medullary cavities of bones, has found, shortly afterwards, the metallic globules dispersed through the lungs and liver of the animal. After all fatal amputations, which have been followed by the formation of secondary abscesses, the membrana medullaris of the divided bone should be examined; in many cases it will be found inflamed, and otherwise changed, and its veins filled with pus.

Cruveilhier assures us that the commencement of the mischief, when it occurs after injuries of the head, may often be detected in the diploe of the skull. We are not always to expect to find the lining membrane of a vein inflamed, when its cavity contains purulent matter; and, if it be inflamed, we are to remember that the inflammation may have been subsequent to, and indeed caused by, the presence of the morbid fluid in its cavity. Pus, therefore, may exist in a vein, without being the product of a local phlebitis; and the only rational explanation of this occurrence, is to suppose that it has been absorbed directly from some abscess, or purulent deposit, or that it has been generated by the decomposition of the contained blood, which has become arrested at the part. That blood at rest, whether within a bloodvessel, or which has been extravasated into the cellular tissue, may become converted into a purulent fluid, has been satisfactorily demonstrated by Gendrin and others; and we have already alluded to the occurrence of pus in the centre of coagula within the cavities of the heart. It deserves to be noticed, that venous blood has a much stronger tendency than arterial to the change we are alluding to.

Admitting that pus may be absorbed directly from an abscess into the adjacent veins, we arrive at another difficulty in the pathological history of secondary abscesses—to explain in what manner the absorbed pus acts in giving rise to these deposits. Dr. Nasse has made many experiments on the lower animals—by injecting pus into their veins, by fracturing their bones, and by retaining suppurating wounds open for a length of time, with the hope of throwing some light on this curious subject, but hitherto quite in vain. It is a point of dispute, whether secondary abscesses are ever formed without being preceded by inflammation in the part. The opinion of the older surgeons was, that there is a direct metastasis of the matter, and, therefore, that the abscesses may be quite independent of any inflammatory process. This opinion has been adopted by several of the best modern pathologists, as Chaussier, Marjolin, Ribes, and Velpeau—all of whom positively deny the local or inflammatory origin of secondary abscesses. The reasons which they adduce in favour of their opinion are the following: the absence often of any traces of inflammatory action in the nighbourhood of the abscesses, the rapidity with which the purulent deposits sometimes take place, the circumstance of their being dispersed through an organ while the intervening tissue is not sensibly diseased—not to mention the obscure and indefinite symptoms which accompany their generation during life.

These arguments, although strongly opposed to the doctrine of local inflammation as being the cause of secondary abscesses, do not warrant our immediate assent to the rival doctrine, of the metastasis of the purulent matter itself, from one part of the body to another at a distance. How are we to explain the exsudation, or secretion of the large globules of pus from the minute capillaries of the affected part? And why does such an exsudation or secretion, if it is practicable, take place at numerous circumscribed spots in one or more organs at the same time? The prudent pathologist will hesitate, we think, to regard the development of secondary abscesses, as the effect of a mere mechanical translation of a fluid from one part to another.

The advocates for the inflammatory origin of secondary abscesses are numerous and respectable. Morgagni, Boyer, Rose, Lawrence, and Cruveilhier, may be enumerated among them.

Dr. Nasse agrees, to a certain extent, with those pathologists who regard the formation of secondary abscesses as the result of an inflammation developed in the parts affected—of an inflammation however not simple and uncomplicated, but accompanied, if not actually induced by, a purulent vitiation of the circulating fluid. He supposes that, by the admixture of purulent matter with the blood, the latter becomes contaminated, and has a stronger tendency than in health to the deposition of its fibrinous portion. If the poisoning of the blood by the virus of glanders can, without any primary local phenomenon of disease, induce abscesses in different parts of the body, and if imperfect nutrition, especially when this is asssociated with exposure to damp and cold, can generate a tendency to the formation of purulent deposits, what reason is there for wonder that a depravation of the sanguineous fluid from other causes may give rise to the same or similar effects? It is a well-known fact that, in cases of phlebitis, venæsection performed at any part, however remote from the seat of the inflamed vein, is apt to be followed by an attack of phlebitis in the wound. Now this fact accords with the general position that diseases, in which there is any depraved assimilation of the blood, have a marked tendency to induce, upon the slightest and often without any appreciable cause, a similar morbid state in other parts distant from the seat of the primary lesion. This is the case with tubercles, cancer, fungus hæmatodes, and probably also with any local admixture of pus with the blood. We cannot indeed discover any cancerous or medullary matter in the blood, yet we know that there is a strong tendency to the development of the cancerous, &c. formative action in many parts of the body, near to, as well as remote from, the seat of the primary disease. Now, if we apply the same mode of reasoning to phlebitis, we may find a rational explanation of secondary abscesses without adopting the notion of a positive admixture of pus in the whole circulating mass, or of a direct metastasis of the pus from one region of the body to another. How the vitiation of the blood probably acts in giving rise to the development of secondary abscesses will perhaps be best understood by having our attention directed to the post-mortem appearances which, at the commencement of this paper, we indicated as appertaining to the first stage of their formation. It was there stated, that the early or incipient structural changes, as observed in the lungs and liver, consist in congested and discoloured patches, which have a great resemblance to parts in which there had been some time before an infiltration of blood. The form of these patches is generally sharply defined, in consequence probably of the morbid change affecting only one lobule or portion of parenchyma, to which a particular venous branch appertains. The cause of this congested state of distinct lobules or portions may be either an obstruction of the blood in the capillaries, or an absolute extravasation of it into the parenchymatous tissue—the former conjecture is the more probable. Now this obstruction we are disposed to attribute either to an inflammatory action, or to the coagulation of the blood in the small vessels, induced, it may be, by the unhealthy condition of this fluid.

If the blood is once obstructed in the capillaries, coagulation, to a greater or less extent, will take place in the neighbouring veins, and thence extend to the larger ones; this tendency to coagulation being greatly increased by the vitiated state of the blood itself. With or without inflammation of the lining surface of the veins, the contained blood becomes gradually converted into a puriform fluid.

This theory of the congestion of the blood-vessels, and of the subsequent formation of purulent matter in the affected part, does not apply so well to the case of the synovial membranes (often the seat of secondary suppurations) as to that of the parenchymatous viscera. It is to be remarked, however, that such articular suppurations are almost always attended with much more severe pain, and with more decidedly inflammatory symptoms, than secondary suppurations in other parts of the body. The character or type of the inflammatory action is very different indeed from ordinary arthritis—it has a peculiar disposition to terminate rapidly in purulent effusion.

M. Boyer, in one of the earlier numbers of the Gazette Medicale de Paris, has proposed a chemical rationale of the constitutional symptoms which usually attend the formation of secondary abscesses. He suggests that it is the ammonia contained in the pus, which exerts a noxious influence on the system. He found that pus, which has been purified by means of chlorine, may be injected into the veins of an animal with much less injurious consequences than when it has not been so purified. He recommends strongly the internal and external use of chlorine in typhus and in all diseases where the condition of the blood is suspected of being vitiated from the admixture of impure animal fluids.

Although friends to the adoption of a modified humoral pathology and of a cautious humoral system of therapeutics, we are not inclined to give our assent to the doctrine propounded by Boyer for the etiological explanation of secondary abscesses.—*Rust's Magazin für die gesammte Heilkunde.*

---

REMARKS ON THE PROXIMATE CAUSE OF GOUT. By Dr. WENDT, of Breslau.

To determine the essential characters, and genuine nature of gout, is much less easy than many physicians of the present time are inclined to believe. That it is a disease "totius corporis," not confined to one part or one system of the body, but affecting, although unequally, all parts of the body; that it is usually a sure sign or indication of an inflammatory state of the constitution, that its most common seat is in the smaller joints; that it is apt to induce in course of time a more or less abnormal state of the fluids, and a tendency to various lesions of the solids; —these are characters which make up a general and comprehensive description of gout, and probably a more correct one than can be conveyed in any short sentence, or condensed definition. In the excessive ardour of medical reform, some physicians of the present day have boldly announced that gout is nothing but simple arthritis or inflammation of the joints. This is a most unwise and dangerous doctrine: for, on the one hand, gout may exist without even affecting any articular cavity, and on the other, there may be the most severe arthritis without the slightest trace of genuine gouty disease being present.

In investigating the nature of gout, it is of importance ever to keep in mind that this is one of the diseases which may be truly and strictly congenital and hereditary, and therefore that certain persons have a peculiarly strong predisposition to its attack in some modification or another. This consideration alone might lead us to believe that the elements or germs of the disease are deeply and intimately interwoven with the frame, and that the ordinary existing causes of inflammation will not be sufficient to induce an attack of gout, unless the system of the patient be at the time predisposed to the specific morbid state or cachexia, with which it is always associated. Exposure to cold and damp are often sufficient to excite a paroxysm of gout, but never, be it remembered, in a person whose habit of body is perfectly sound and healthy at the time.

True therefore it is, that "neither simply checked perspiration is ever sufficient to cause gout, nor will the most complete diaphoresis ever cure it."

Patients have often been kept drenched in profuse perspirations for several days continuously, with but little relief to their distress, *unless the perspiration smells chalky*, or until Nature rids herself of the peccant matter, which is offending her. Cullen's doctrine that gout is dependent upon weak digestion has been, with various modifications, adopted by many of our modern physicians. Some have insisted much upon a disturbance of the power of chylification, and a consequent derangement of the assimilation of the blood, as invariable precursors of gout. But this is not strictly correct; and that admirable authority, Sydenham, has with great propriety described an 'arthritis fortiorum'—a form of the disease which indicates great activity of the digestive powers, and a plethoric and vigorous state of the body.

How often it happens that just before a gouty paroxysm, the patient has been in most robust health; eating, digesting and sleeping well, and presenting no irregularity or any symptom of approaching disease, except perhaps that the urine is scanty, turbid, and of a deep colour, and strong acid smell. This is the delusive state, which Sydenham has aptly compared to the 'sunshine before the storm.' Weakness of digestion cannot surely be admitted as the exciting cause of gout, in such a case as this.

To understand the true nature of gout correctly, it will be necessary to make some general remarks on the cachexiæ, or diseases which are dependent upon some irregularity or disturbance of the assimilative and nutritive powers of the body.

Some cachexiæ originate from an abnormal condition of the blood (blutmischung); such as icterus, chlorosis, and scorbutus; others depend upon an unhealthiness of the white juices, and to this section may be referred dropsy, gout, &c. Whatever be the form of gout, we believe that the diseased action originates and consists in a tendency to the formation and the elimination of earthy matter from the system. This is in fact the 'causa efficiens morbi.'

The development and elimination of the earthy portion from the general mass of the nutritive juices, and the tendency which this has to become deposited in particular textures—these are the essential and invariable elements of gouty disease. The peculiar liability of the joints to be affected with gouty inflammation and depositions is owing probably to the circumstance of the earthy elements being held more loosely in the joints than in the other parts of the osseous system.

That gout at its commencement is usually attended with an inflammatory diathesis, is not remarkable, seeing that vascular excitement accompanies some of the forms or stages of other cachexiæ, as for example the 'hydrops calidus,' the 'chlorosis florida,' and the pyrexial periods of phthisis.

The various forms or modifications of gout, although all primarily and essentially originating in the same morbid state, viz. that of the formation of earthy matter in the system, depend chiefly on the condition of the nervous and vascular excitabilities at the period of the attack. Perhaps the most practically important division of the disease is into the two species, 'arthritis florida, sive fortiorum,' and 'a. atonica, sive debiliorum.' Gout may be either localized—the a. organica—or it may be flying about,—the a. vaga. When the gout-formation fixes upon any of the nobler parts, it is called 'a. retrocessa;' and that condition, in which the process of earthy formation is going on, and in which there is consequently an excess of earthy elements in the body, without however any local deposition, or established inflammatory action, may be designated the 'a. retenta.'

The gouty dyscrasis, when it falls chiefly on the eyes, is apt to induce cataract; when the valves and blood-vessels of the heart are the parts chiefly affected, angina pectoris or other forms of disturbed circulation and breathing will be induced; and the kidneys, when attacked with gouty disease, are apt to secrete an excessive quantity of uric acid and usually become inflamed, and in course of time disorganized. These forms of gout may alternate with, and they may supersede, and as it were, neutralize each other. A striking example of this occurred in the case of an old gentleman under Dr. Wendt's care. He had for several years been subject to severe attacks of angina pectoris, which had been gradually increasing to such a severity, that his friends were often expecting to hear of his sudden death.

By a fortunate translation of the morbid process, renal calculi were generated, and, in the course of a few weeks, every symptom of stenocardia vanished completely.

In all gouty metamorphoses, the tendency to earthy deposition is more or less appreciable, but most distinctly so when arthritic abscesses are formed. Sanctorius alludes to the contents of such abscesses in these words: "phlegma gypseum molle liquidæ calci simile." Dr. Wendt relates the following striking examples. In two very gouty patients, large quantities of a white fluid, very like an emulsion of chalk, were discharged from the wrist-joints, which had a short time previously suppurated and burst. When carbonic or phosphoric acid was added to this white discharge, it became thick and almost concrete. The abscesses healed, and afterwards opened again, affording a similar white discharge to the former one. When there is no distinct deposition of gouty matter, in the joints or other parts, traces of earthy matters may very generally be detected in many of the secretions and excretions, and more especially in all critical discharges The urine and the saliva, it is well known, are often, in these circumstances, loaded with earthy matter: hence the very common tendency to the formation of urinary calculi, and of incrustations about the teeth. In many cases, the perspiration has a distinct chalky smell (nach kalk riechende schweiss). Dr. Wendt alludes to a case where this phenomenon was very appreciable.

A patient who had long suffered from stenocardia (angina pectoris), was seized with a severe attack of arthritis, and during the continuance of the joint affections, all the thoracic distress vanished. During this very favourable "reconstruction" of so dangerous a malady, there had been a profuse perspiration, and it was noticed by all the attendants, that the perspiration had a chalky smell (kalkartigen geruch). Many of the features of gout indicate that it is a disease connected with an irregular and unhealthy (whether from a mere excess, or from a change in properties) condition of the earthy elements of the body, and with a separation of these elements from the general mass of the organization. Not to mention again the characters of actual gouty deposits and of critical evacuations, we may allude to the extreme obstinacy, in general, of the disease—the difficulty of curing it, and the marked tendency which it has to return, even although it may have been superseded, as it were, for a time by some vicarious disease, such as hæmorrhoids or leucorrhœa. As age advances, the disposition to gouty disease certainly increases: perhaps this may be attributable to the greater tendency to the formation of earthy matter in the system, and to the lessened activity of the eliminating organs.

Dr. W. alludes to the old, and still popular belief, that gout is, on the whole, rather favourable than otherwise to longevity. He is not inclined to go quite so far as Erasmus and Cardanus, and write an eulogy on gout; but often (says he) he has had occasion, in his own practice, to remark the number of grey-haired veterans among the "podagrists"! The diseases to which they most frequently fall a sacrifice are angina pectoris, hydrothorax, asthma, and phthisis pituitosa.

The treatment of gout by the best German physicians is, we are informed by Dr. Wendt, directed rather to prophylactic than to directly curative means. A severe paroxysm of gout may be mitigated, and perhaps shortened—but it cannot be arrested, or suddenly cut short. The proper termination of each paroxysm is denoted by the elimination of the superfluous earthy matter, either in the form of gouty deposits, or carried off by some critical evacuation. Wisely has Sydenham cautioned the physician against overofficiousness in trying to quiet the pains of gout: the pains, says he, are ' amarissimum Naturæ pharmacum."

The most important indications are, to assist Nature in relieving herself of the peccant matter which is annoying her—in correcting the morbid diathesis —in the removal of all complications which may supervene, and in counteracting any tendency to seizures of internal organs.

There cannot therefore be any uniform, far less any specific treatment of gout. It must be modified and regulated by the circumstances of the case, by the constitution of the patient, and by the curative indications which Nature may happen to point out. Dr. W. says that as for himself he has never yet *cured* a severe case of genuine gout: the paroxysm ceased, when the product of the dyscrasis was separated from the system, and all that he was ever able to do, was simply to employ prophylactic means against future attacks. Whatever tends to preserve the healthiness of the processes of assimilation and sanguification, (die normalität der mischungsverhaltnisse), and protects and promotes the firmness and solidity of the tissues, will assist materially in fortifying the body against the gout. A well assorted diet may do much; but regular and regulated bodily exercise will do more.

Gout is truly a disease of towns; it is but little known in the country, except by those who carry thither the luxury and idleness of town lives. Some old English author has aptly remarked that 'gout is the disease of those who will have it.' Even those who are strongly predisposed to it, seldom suffer from its attacks, as long as they live an active and an abstemious life. Dr. Wendt powerfully recommends the use of the vapour-baths, as used so commonly in Russia. Some of his countrymen have lately proposed to treat gout by making the patients drink enormous quantities of hot water, with the hope of fairly *washing out* the peccant matter from the system. Dr. W. very judiciously prefers the use of *external,* we that of *internal* hot baths.—*Rust's Magazin für die gesammte Heilkunde.*

---

### Staphyloma treated by Excision of the Cornea, and not followed by Destruction of the Eye.

A young girl had suffered from a severe attack of purulent ophthalmia of the right eye, which had been followed by complete staphyloma. The cornea was quite opaque throughout, and very prominent, so that the eyelids could not close over it, when the patient was asleep. Numerous enlarged vessels extending from the sclerotic coat ramified on the surface of the marbly-blue cornea. As is usual in cases of staphyloma, this little girl had got into the the habit of stooping forwards, and leaning the head towards the affected side.

Dr. Buck of Lubeck, under whose care she was placed, recommended that the projecting part of the cornea should be cut off; expecting that by this operation the contents of the eyeball would be discharged, and that the patient might then wear an artificial eye. The second eye having been closed, Dr. B. with one sweep of a cataract knife, sliced off the cornea to within a line's breadth of its attachment to the sclerotic.

The aqueous humor with some drops of blood flowed out; and immediately afterwards the eyelids closed together with a spasmodic firmness, so that nothing else escaped. On examining the detached cornea, it was found to be of a leathery consistence; its inner surface was as smooth as its outer, and therefore there could have been no adhesion of the iris to it, as is the case most frequently in cases of staphyloma.

The patient was kept quiet for a fortnight in a dark chamber. At this time on removing for the first time the bandage from the sound eye, a sharp pain was immediately felt in the diseased one.

By the end of the third week, she could open both eyes without any annoyance, and then it was first discovered that a membrane of plastic lymph had been formed in the place of the removed cornea. This membrane was, as might have been expected, flat, and being of a blueish white colour, the iris could not be easily distinguished through it. The adhesion of this membrane to the edge of the remaining portion of cornea was so close and intimate, as to be not distinctly recognizable.

After some months, the patient's power of vision was somewhat greater than it had been before the operation. She could distinguish, though imperfectly, the profile of large dark objects placed before her. The form of the eye-ball, with the exception of its anterior face which was permanently flat, remained unaffected.—*Rust's Magazin.*

---

### Contagion of the Plague.

M. Clot Bey, the distinguished surgeon of the Pacha of Egypt, has addressed a letter to M. Chervin, (who is one of the most vehement of anti-contagionists,) on the plague, which raged at Alexandria from Nov. 1834 to March of last year. During that time upwards of 20,000 persons died from it. M. Clot observes, that almost all the medical men in Alexandria and Cairo are decided contagionists, and are so much impressed with fears of the communicability of the plague by contact, that when they visit their patients, they put on dresses of oiled silk, carry long sticks in their hands, and examine the sick at some distance from the bed-side. They do not believe that the plague is ever diffused by merely epidemic agency. Other physicians, less afraid, " ne s'encapuchonnent pas;" but they avoid touching the skin of their patient, and feel the pulse either through a tobacco-leaf, or after having wetted their fingers with oil, or vinegar; while a third set, uncertain what to think, and more hazardous than the rest of their brethren, do not take any further precautions, than what are usual after visiting cases of typhus and other fevers. The plague, of which M. Clot speaks, appeared at Alexandria in November, and in Cairo towards the end of December; but it had not assumed an alarming character, until within a fortnight previously to his writing to M. Chervin, about the middle of March.

The prevailing belief among the professional men, as well as among the public in general, was that the pestilence had been imported. This might have been the case, but certainly it was not satisfactorily proved; and as for myself, (M. Clot) I must confess that I am inclined to attribute the invasion rather to epidemic influences, than to the direct introduction of any infected fomites.

M. Clot admits that some indeed of the facts which he witnessed tended to shew that the disease may be transmitted from one person to another; but that the mass of them was decidedly unfavorable to this idea. He alludes to the case of himself and of five other medical men, who were in the daily habit of touching and examining the sick, of spending several hours at their bed-sides, and of dissecting the corpses in a small contracted place.

The medical students also and the nurses were in frequent, nay, even continual intercourse with the sick, and yet "jusqu'à present aucun accident n'a en lieu." Another argument may be drawn from the circumstance that the disease did not break out at Cairo, for nearly two months after its appearance at Alexandria, although there was a constant communication between these two towns. The crews too of some of the ships in the bay of Alexandria were attacked with the disease, although all intercourse with the shore was most rigorously prevented.

In conclusion, M. Clot observes that the sanitary regulations which were enforced, appeared to him rather to promote than to obviate the spreading of the pestilence, not to mention the very unnecessary interruption which they caused to traffic, and business.—*Archives Gen.*

